# Mapping spatial distribution and geographic shifts of East African highland banana (*Musa* spp.) in Uganda

**DOI:** 10.1371/journal.pone.0263439

**Published:** 2022-02-17

**Authors:** Dennis Ochola, Bastiaen Boekelo, Gerrie W. J. van de Ven, Godfrey Taulya, Jerome Kubiriba, Piet J. A. van Asten, Ken E. Giller

**Affiliations:** 1 International Institute of Tropical Agriculture (IITA), Kampala, Uganda; 2 Wageningen University and Research (WUR), Wageningen, The Netherlands; 3 National Agricultural Research Laboratories (NARL), Kawanda, Uganda; Potsdam Institute for Climate Impact Research, GERMANY

## Abstract

East African highland banana (*Musa acuminata* genome group AAA-EA; hereafter referred to as banana) is critical for Uganda’s food supply, hence our aim to map current distribution and to understand changes in banana production areas over the past five decades. We collected banana presence/absence data through an online survey based on high-resolution satellite images and coupled this data with independent covariates as inputs for ensemble machine learning prediction of current banana distribution. We assessed geographic shifts of production areas using spatially explicit differences between the 1958 and 2016 banana distribution maps. The biophysical factors associated with banana spatial distribution and geographic shift were determined using a logistic regression model and classification and regression tree, respectively. Ensemble models were superior (AUC = 0.895; 0.907) compared to their constituent algorithms trained with 12 and 17 covariates, respectively: random forests (AUC = 0.883; 0.901), gradient boosting machines (AUC = 0.878; 0.903), and neural networks (AUC = 0.870; 0.890). The logistic regression model (AUC = 0.879) performance was similar to that for the ensemble model and its constituent algorithms. In 2016, banana cultivation was concentrated in the western (44%) and central (36%) regions, while only a small proportion was in the eastern (18%) and northern (2%) regions. About 60% of increased cultivation since 1958 was in the western region; 50% of decreased cultivation in the eastern region; and 44% of continued cultivation in the central region. Soil organic carbon, soil pH, annual precipitation, slope gradient, bulk density and blue reflectance were associated with increased banana cultivation while precipitation seasonality and mean annual temperature were associated with decreased banana cultivation over the past 50 years. The maps of spatial distribution and geographic shift of banana can support targeting of context-specific intensification options and policy advocacy to avert agriculture driven environmental degradation.

## Introduction

East African Highland Banana (*Musa acuminata* genome group AAA-EA; hereafter referred to as banana) provides food and income for over 30 million inhabitants of the African Great Lakes Region [1]. The plant’s asynchronous fruiting habit allows farmers to harvest throughout the year [2], providing a continuous supply of food in contrast with seasonal crops. Also, banana provides soil cover to control soil erosion on hilly landscapes [3]. Uganda has been the leading producer of banana in Africa for over five decades [4], although current yields of 5 to 30 t ha^-1^ yr^-1^ are well below attainable yields of more than 60 t ha^-1^ yr^-1^ [5, 6]. Several abiotic and biotic factors are underlying causes of the yield gap of banana on smallholder farms in Uganda. Nutrient deficiencies combined with drought stress are the primary abiotic constraints [6, 7], with potassium and nitrogen deficiencies accounting for up to 68% of the banana yield gap [8–10]. Banana is also affected by pests, notably banana weevil (*Cosmopolites sordidus*) and nematodes (*Radopholus similis; Pratylenchus goodeyi*) [11–13], and by diseases such as Xanthomonas wilt (*Xanthomonas campestris* pv. *musacearum*), Fusarium wilt (*Fusarium oxysporum* fsp. *cubense*) and Black Sigatoka (*Mycosphaerella fijiensis*) [14, 15]. The effects of these constraints are exacerbated by poor agronomic management [6, 7]. Interventions to address these constraints need to be tailored to the biophysical conditions and socioeconomic opportunities that vary across agroecological zones.

Wortmann and Eledu [16] used county-level agricultural, economic, demographic, climatic and soil characteristics for delineating and defining Uganda’s 14 agroecological zones (AEZs). The differences in acreage of staple food crops (banana, maize (*Zea mays* L.), cassava (*Manihot esculenta* Crantz), sweet potato (*Ipomoea batatas* [L.] Lam.), potato *(Solanum tuberosum* L.*)*, finger millet (*Eleusine coracana* Gaertn.), common bean (*Phaseolus vulgaris* L.), groundnut (*Arachis hypogaea* L.), sorghum (*Sorghum bicolor* [L.] Moench), and rice (*Oryza sativa* L.)) across the country highlight differences in biophysical suitability and socioeconomic importance of specific crops across AEZs. The principal AEZs for banana are characterised by even rainfall distribution with less than three dry months each year [17, 18]. A geographic shift in banana coverage has been reported with reductions in traditional growing areas in the central region and expansion in the southwestern region [19]. In some districts of the eastern region, annual crops (i.e., cassava, sweet potatoes, common bean, maize, and groundnut) have replaced banana [20]. However, the magnitude of change and underlying drivers of these observed changes are yet to be determined.

We set out to map the current distribution of banana in Uganda and to understand how this has changed over the past five decades. Our specific objectives were to i) map the current distribution of banana in Uganda, ii) identify approaches for predicting the spatial distribution of banana using remote sensing data, iii) determine biophysical factors that influence the spatial distribution of banana, iv) assess the geographic shift in banana production areas, and v) evaluate the biophysical factors associated with geographic shift in banana distribution over the past five decades.

## Materials and methods

### Banana-based cropping systems in Uganda

Banana-based cropping systems in Uganda consist of landscapes where thick banana groves extend from the lowlands and rise onto upper slopes and hilltops. Banana cultivation is particularly important in the Lake Victoria region, Mount Elgon, the interior plateau south of Lake Kyoga, the Ankole ridges and downlands, and the rift valley shoulder [21]. This staple food crop is cultivated by resource-poor and risk-averse farmers who use traditional farming methods and have poor access to quality fertilizers to improve soil fertility or crop protection inputs to reduce crop losses to pests and diseases. The percentage contribution of bananas to the total food acreage demonstrates the importance of the crop. Often farmers mix multiple cultivars of cooking bananas (‘Matoke’), beer bananas (‘Mbide’), roasting bananas (‘Gonja’), and dessert bananas (‘Ndiizi’ and ‘Bogoya’) within fields, farms or across landscapes [22]. However, the cooking type banana dominate the mixed stands across the country.

### Digitizing the historical map of banana

**Fig 1:**
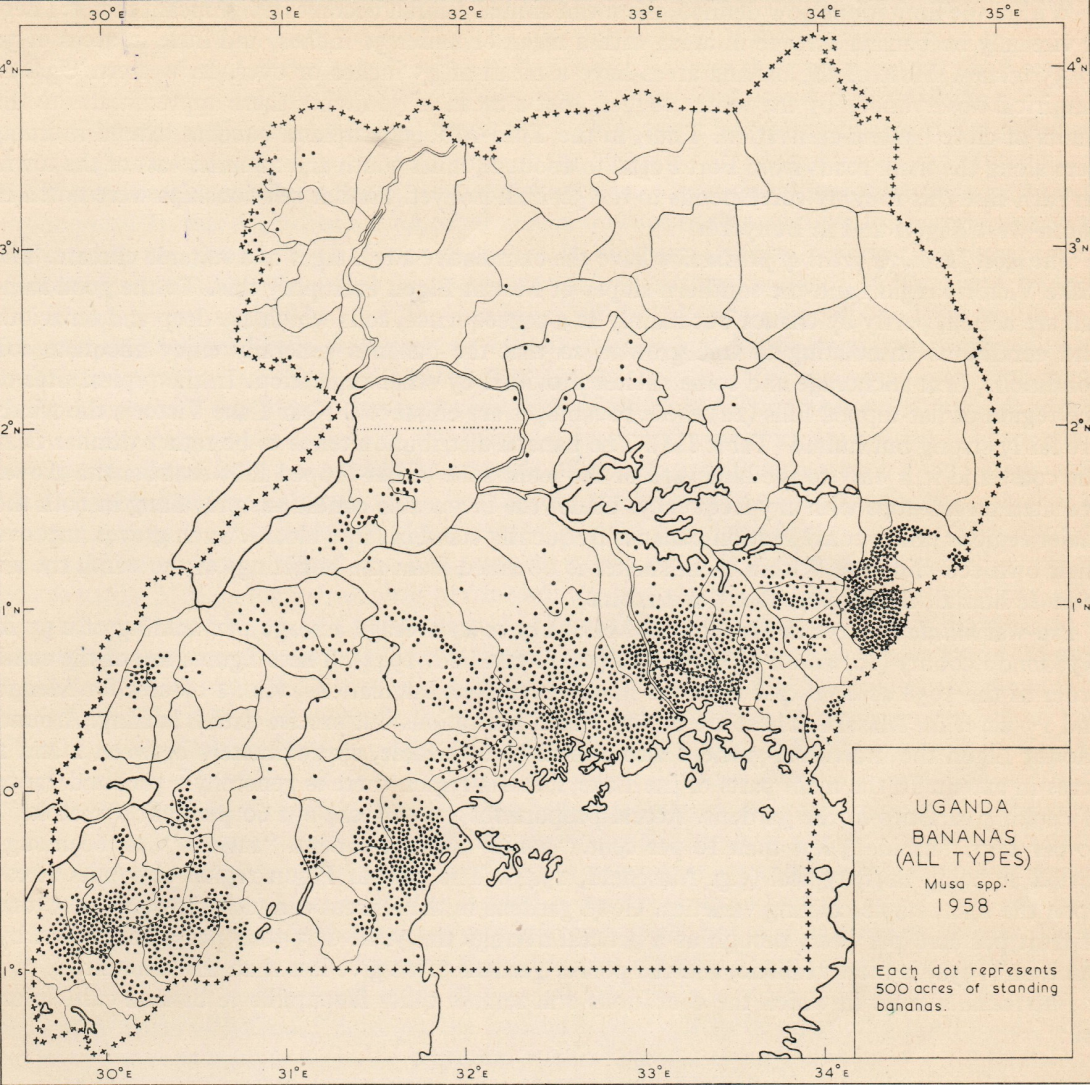
Scanned map of historical locations with banana in 1958. Each dot represents 2.02 km^2^ of standing banana. A total of 2400 dots are spread across Uganda equivalent to 4,856 km^2^ of banana. Image reproduced with permission of Giles Clark, the copyright holder.

A hand-drawn map from 1958 [21] depicting banana areas using a dot equivalent to 2.02 km^2^ (500 acres) with a total of 2400 dots or 4,856 km^2^ across Uganda (Fig 1) was digitised in two steps. First, the scanned map was processed into a black and white image. Georeferencing was performed using the Georeferencer plugin in Quantum GIS (QGIS) version 2.18.13 for Mac [23]. A series of positions on the black and white image were identified that corresponded to positions on the current administrative boundaries of Uganda. These ground control points were used for matching *x* and *y* pixel coordinates on the image with the longitude and latitude coordinates. A second-order polynomial transformation and nearest neighbor resampling were used to georeference the image to Universal Transverse Mercator (UTM) zone 36N. Borders were manually erased from the georeferenced image using the GNU Image Manipulation Program (GIMP) version 2.10.2 for Mac [24] resulting in an image that retained only the dots representing banana locations. Second, the dots were vectorised, geometries cross-checked, and anomalies fixed. Centroids of the dots were determined and buffered with 800 m, thereby creating vectorised circles with areas matching the original dot areas of 2.02 km^2^ presented in Fig 1. The hand-drawn nature of the 1958 banana distribution map resulted in a mismatch between boundaries of the georeferenced image and the Global Administrative Areas (GADM) boundaries. Visual inspection revealed that average boundary deviation was between 1 and 2 km, rarely more than 5 km. This meant that 1:1 overlay of the historical (1958) and latest (2016) banana distribution maps would be unreliable. Hence, the nearest accuracy that we could achieve for the 1958 distribution map was to superimpose a vector grid of 5 km × 5 km on the vector layer of circles to account for the 1–5 km deviations, with each grid cell indicating the cover of banana area inside.

### Geosurvey presence/absence data

**Fig 2:**
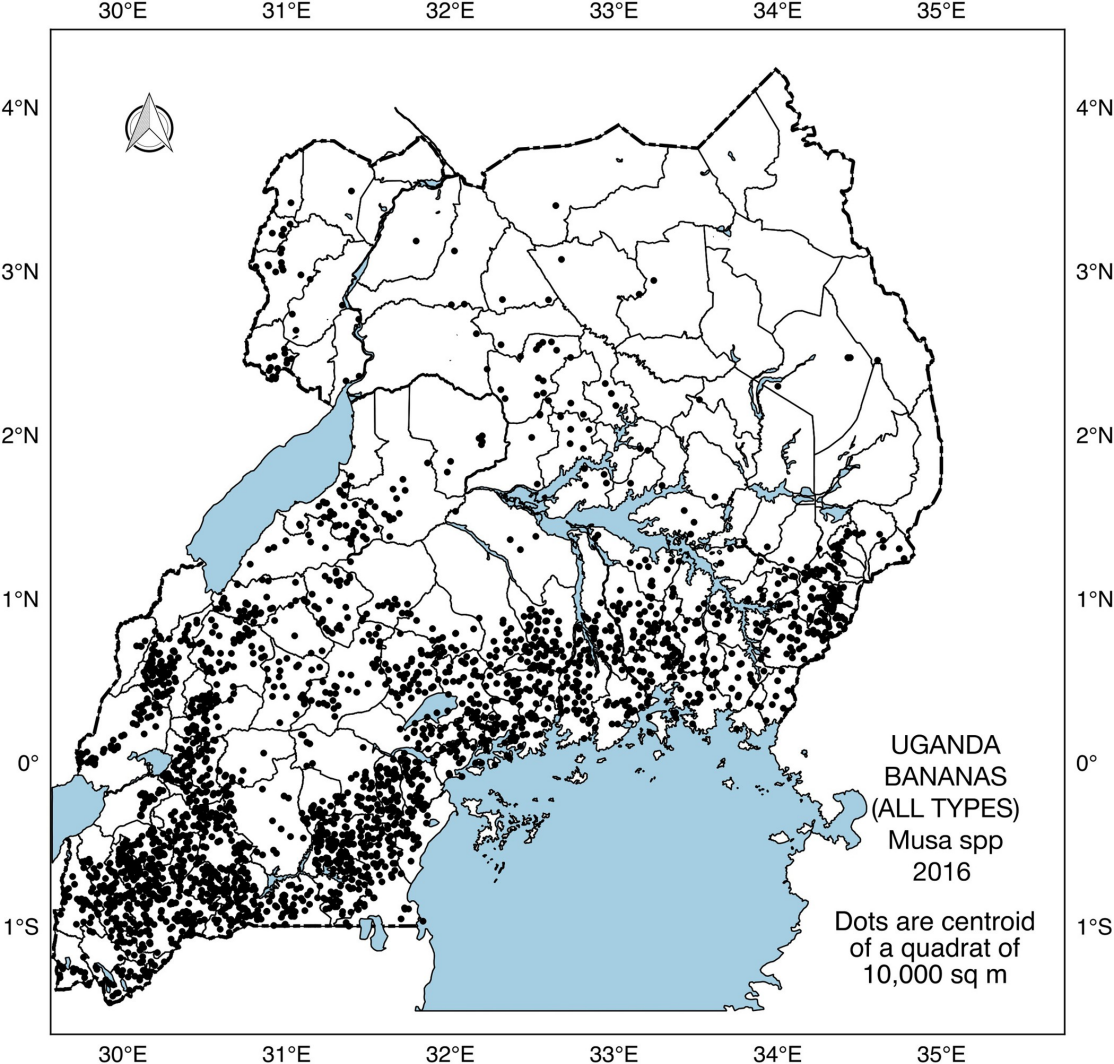
Sampled locations with banana in 2016. Each dot represents the centroid of a quadrat of 10,000 m^2^ used for collecting banana presence/absence information during the Geosurvey. Data acquired with permission of Markus Walsh, Africa Soils Information Service (https://doi.org/10.17605/OSF.IO/J8Y3Z).

Geosurvey is an online platform that facilitates the collection of geospatial data through assessment of high-resolution satellite imagery [25]. A team of 15 analysts examined images of randomly chosen locations within Uganda and determined the presence/absence of banana inside a 100 m × 100 m quadrat. Google Earth is the default image option for the Geosurvey platform, but analysts can switch to alternative image sources (i.e., Bing Maps, DigitalGlobe, PlanetLabs, and MapBox) whenever cloudiness obstructs the visibility of the land surface for a given image. This resulted in a dataset of 19,130 banana presence/absence observations. Of these, 181 observations were invalid because 174 observations fell outside the boundaries of Uganda and 7 observations were duplicates. Consequently, the sampled locations were reduced to 18,949 comprising 16,522 absence and 2,427 presence of banana (Fig 2).

### Point filtering to remedy spatial dependence

The proximity between presence/absence points means the biophysical conditions at these locations would probably be more related than if the points were distant from each other. Point clustering or dispersal invalidates the assumption of spatial independence, which is vital for unbiased predictions. Hence, spatial filtering was performed to attain the required random point pattern. First, we created 5 km × 5 km pixels to facilitate a quadrat count of unique presence/absence points. The polygon of unique point counts aided random sampling of points, so that no more than one point remained within a 5 km × 5 km pixel. Second, we determined point patterns through average nearest neighbor analysis executed using the ArcGIS Desktop version 10.6.1.9270 [26]. The approach involves measuring distances between points and their nearest neighbors and compares them against a hypothetical random distribution. A clustered or dispersed pattern exists if the observed mean distances are less or greater than the expected mean distance of the hypothetical random distribution, respectively. A positive or negative average nearest neighbour index and statistically significant *z*-score or *p*-value indicate a less than 1% likelihood that a clustered or dispersed pattern in the dataset results from random chance, respectively.

### Gridded covariates and multicollinearity

Spatially continuous data that are readily available on the internet were acquired. The 71 remotely sensed and gridded covariates consisted of 21 climatic, 19 edaphic, 19 vegetation, 6 socio-economic, and 6 topographic variables. Respective covariates, sources, resolutions, and time frames are presented in the S1 Table. All covariate variables were resampled to 250 m resolution, consistent with the global gridded soil information [27]. The covariates were centered (mean minus values) and scaled values divided by standard deviation) to ensure zero mean and unit variance using the R package raster [28]. We determined an optimal number of independent covariates because multicollinearity among covariates could affect model stability and approximation quality [29]. For this purpose, we employed two approaches for selecting relevant and independent covariates, i.e., recursive feature elimination and subjective feature selection.

### Approaches for selecting independent relevant covariates

#### Recursive feature elimination with resampling

**Fig 3:**
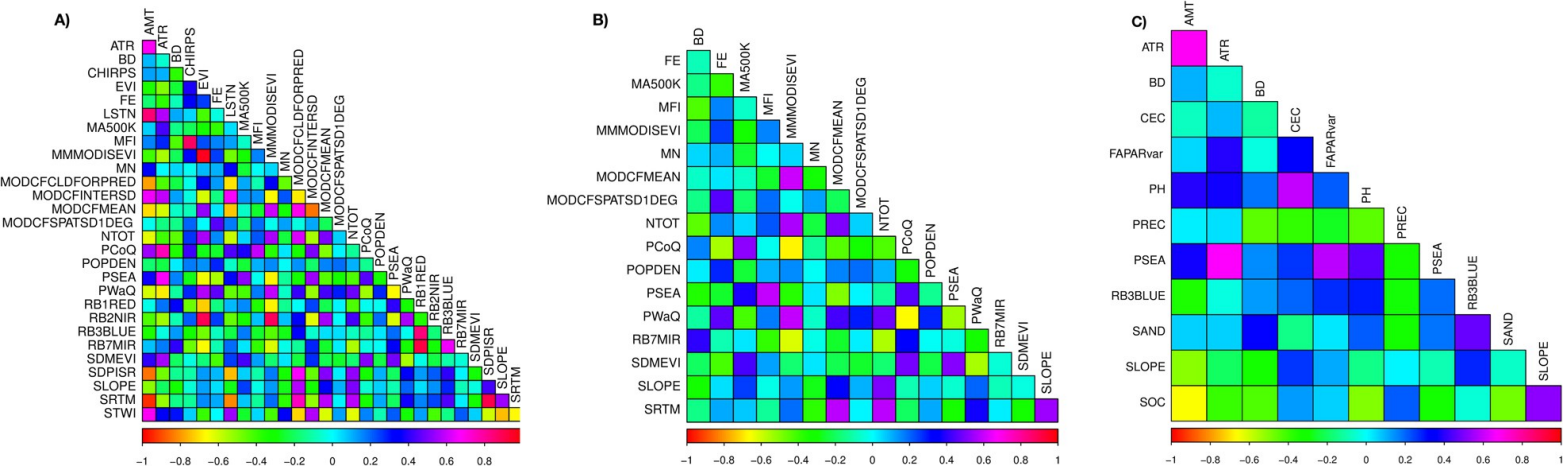
Correlation among selected covariates A) 29 covariates after recursive feature elimination; B) 17 covariates with Pearson’s correlation coefficient (r) less than ± 0.7; C) 12 covariates selected using a subjective approach.

Recursive feature elimination is a wrapper algorithm that assesses multiple covariate subsets while iteratively removing the weakest covariates until the optimal number of covariates is reached [30]. The algorithm uses ten-fold cross-validation to score the covariate subsets and select the best ranked collection of covariates that maximises model performance. Applying this approach led to the selection of 29 covariates that describe the biophysical and socio-economic environments of banana in Uganda. Visual inspection of the correlation matrix of the 29 covariates was done to identify and eliminate covariates with correlation coefficient (r) greater ± 0.7 [31]. Consequently, 17 uncorrelated covariates were retained for machine learning prediction (Table 1, Fig 3).

**Table 1:** List of 29 covariates selected from a list of 71 variables using recursive feature elimination and further selection of 17 uncorrelated covariates (shaded grey).

Covariate type	The 29 selected covariates	The 17 uncorrelated covariates	References
Climatic	AMT, Annual Mean Temperature (°C)		[32]
	ATR, Annual Temperature Range (°C)		
	PCoQ, Precipitation of the Coldest Quarter (mm)		
	PWaQ, Precipitation of the Warmest Quarter (mm)		
	PSEA, Precipitation Seasonality (CV)		
	LSTN, Land Surface Temperature Night-time (°C)		[33, 34]
	MFI, Modified fournier index		
	CHIRPS, Climate Hazard InfraRed Precipitation with Station (mm)		[35]
	MODCFSPATSD1DEG, Cloud variability 1-degree (Standard deviation)		[36]
	MODCFINTERSD, Cloud Inter-annual variation (Standard deviation)		
	MODCFCLDFORPRED, MODIS Cloud Forest Prediction		
	MODCFMEAN, Cloud Mean Annual Variation (%)		
	SDPISR, Standard deviation of the potential incoming solar radiation (kWh m^-2^)		[37]
Edaphic	BD, Bulk Density (kg m^-3^)		[27]
	NTOT, Total Nitrogen (g kg^-1^)		
	FE, Extractable Iron (mg kg^-1^)		
	MN, Extractable Manganese (mg kg^-1^)		
Vegetation	EVI, Enhanced Vegetation Index		[38–41]
	SDMEVI, Standard Deviation—Monthly Enhanced Vegetation Index		
	MMMODISEVI, Mean Monthly Enhanced Vegetation Index		
	RB3BLUE, Blue reflectance (Band 3) (nm)		
	RB7MIR, Mid infrared reflectance (Band 7) (nm)		
	RB2NIR, Near Infrared reflectance (Band 2) (nm)		
	RB1RED, Red reflectance (Band 1) (nm)		
Socio-economic	MA500K, Time to Market of 500,000 people (hr)		[42, 43]
	POPDEN, Population Density (persons m^−2^)		[44, 45]
Topography	SLOPE, Slope gradient (%)		[37, 46, 47]
	STWI, Topographic Wetness Index		
	SRTM, Shuttle Radar Topographic Mission Digital Elevation (m)		[48]

#### Subjective feature selection

Biophysical covariates related to light, nutrients, temperature, and water, the factors known to strongly influence crop growth, were selected subjectively from the set of 71 variables. Correlation analysis was then performed to identify and exclude covariates that were strongly correlated (r > ± 0.7). The approach led to the selection of 12 uncorrelated covariates believed to directly affect banana growth and development (Table 2, Fig 3).

**Table 2:** List of 12 covariates selected subjectively and their underlying reasons for their selection.

Covariate type	The 12 selected covariates	Reason for selection	Reference
Climatic	AMT, annual mean temperature (°C)	The annual mean and range of temperature discriminate areas with minimum temperatures below which banana cannot grow.	[49, 50]
	ATR, annual temperature range (°C)		
	PREC, annual precipitation (mm)	Annual amount and distribution of rainfall is important for a perennial crop like banana.	[18, 22]
	PSEA, precipitation seasonality (%)		
Edaphic	pH, soil pH in solution	pH, CEC and SOC are indicators of soil fertility.	[51, 52]
	CEC, cation exchange capacity (cmol kg^-1^)		
	SOC, soil organic carbon (g kg^-1^).		
	BD, bulk density (kg m^-3^)	Bulk density and sand fraction are indicators of rooting conditions, drainage, and soil workability.	
	SAND, Sand fraction (%)		
Vegetation	FAPARvar, fraction of absorbed photosynthetically active radiation–variance (μmol m^−2^ s^−1^)	FAPARvar is indicative of the rate of photosynthesis, while the evapotranspiration rate is quantified using net photosynthesis.	[53]
	RB3BLUE, Blue Reflectance (Band 3)	Blue reflectance helps to eliminate atmospheric noise (cloudiness, smoke) that could affect accurate observation of banana presence or absence.	[54]
Topographic	SLOPE, slope gradient (%)	Slope gradient influences water infiltration versus runoff. Steep slopes are prone to erosive forces that affect nutrient uptake.	[47, 52]

### Extraction of covariate values at sampled point locations

We extracted values from the 12 and 17 independent covariates at the filtered presence/absence locations using the function extract of the package caret [55]. All missing values were omitted since most tree and rule-based models only consider complete information. The resultant datasets were partitioned into training (67%) and testing (33%) using the createDataPartition function of the package caret [55].

### Approaches for prediction and mapping banana

We compared two analytical methods, machine learning and logistic regression, to identify the most efficient approach for predicting the distribution of banana using remotely sensed presence/absence data and multiple independent covariates.

#### Machine learning algorithms

Three machine learning algorithms: random forests (RF), gradient boosting machine (GBM), and neural network (NN) were used to identify patterns in the dataset of banana presence/absence observations related to the selected covariates. Random forests generate an exhaustive number of binary decision trees and allows each tree to cast a random unit vote for the most popular class [56]. The algorithm is computationally effective, a characteristic that is enhanced by the ability to score and rank input features based on their importance. Overall, this embedded dimensionality reduction capability ensures better model stability, reduces risk of overfitting, and increases accuracy of prediction. By contrast, GBM uses an iterative stagewise process whereby weak predictors are sequentially added or removed from the training dataset [57]. A special emphasis is put on learning the instances that were misclassified during the previous training sequence, which decreases the variance of the final prediction. The risk of overfitting is averted by having a smaller learning rate and larger number of trees [58]. Neural networks are adaptive models capable of learning the inherent patterns of the input data by mimicking the neuronal structure of the human brain [59]. The algorithm is particularly effective in handling binary classification problems in which the predictor variables exhibit strong non-linear relationships with the target variable [60]. However, it is critical to scale input values prior to training to prevent the process from converging to the local minimum [61].

#### Training of algorithms

The algorithms were trained with the 12 and 17 covariates. Class imbalance in our dataset meant that trained algorithms could have a bias towards detecting the majority class (banana absence) and a poor recognition of the relatively rare minority class (banana presence). To address this problem, we explicitly specified the subsampling method inside the trainControl function of the package caret [55]. The performance estimates were produced using 10-fold cross-validation repeated five times and default tuning of the relevant hyper-parameters [62]. Performance of the trained algorithms with oversampling (OS), with undersampling (US), and without sampling (WS) was compared using assorted metrics. Accuracy, Kappa, Sensitivity, Specificity and Receiver Operating Characteristic Area Under Curve (ROC AUC) were calculated across resamples by explicitly specifying the defaultSummary and twoClassSummary arguments in the caret trainControl function. ROC AUC was the metric of preference due to its scale-invariant and classification threshold-invariant measure of prediction quality [63]. However, Adjusted F-measure, Brier score, Geometric mean, and Precision Recall Area Under Curve (PR AUC) were also calculated to establish comparative advantage of ROC AUC. Passing the list of trained algorithms to the caret resamples function led to extraction of 50 resampled estimates per calculated metric.

#### Ensemble machine learning

The training outputs of RF, GBM and NN were stacked together to produce an ensemble model that includes a reasonable weighting of each algorithm during prediction using the testing dataset. This helped to compound the variability existing in the training predictions of individual algorithms and thus increase the accuracy of mapping the occurrence of banana in Uganda. The ensemble was fitted using 10-fold cross-validation repeated 5-times. Elastic net regularization of the R package glmnet [64] resulted in a sparse model which allows better interpretation [65]. We evaluated performance of the ensemble models using the metrics Adjusted F-measure, Brier score, Geometric mean, Kappa, PR AUC, and ROC AUC. Statistical comparisons of the ensemble models from training algorithms on 12 and 17 covariates were done using the Wilcoxon rank sum statistic. Probability maps of banana distribution were generated from the ensemble predictions based on algorithm training on 12 and 17 covariates. The ensemble model by default depicted the spatial coverage of banana in terms of the probability of banana presence. The major drawback relates to the several colours in the map which make it difficult to explicitly discriminate the areas with low, moderate, and high coverage of banana. Hence, created ensemble prediction maps were converted from probability to categorical using the probability threshold at which the ensemble model maximises the true positive rate and true negative rate (Max TPR+TNR). We further refined the maps using the SAGA majority filtering tool in QGIS [23].

#### Logistic regression

We ran a separate prediction of banana distribution with the logistic regression model. Banana distribution in the logistic regression model was expressed by the binary response variable with ‘0’ and ‘1’ representing banana absence and presence, respectively.


$$p=\frac{1}{1+{exp}^{-z}}\in [0:1]$$ (1)

$$z={\beta_{0}}_{}+\beta_{1}X_{1}+\beta_{2}X_{2}+\ldots +\beta_{n}X_{n}$$ (2)

Substituting Eq 2 into Eq 1

$$p=\frac{1}{1+\exp[-({\beta_{0}}_{}+\beta_{1}X_{1}+\beta_{2}X_{2}+\ldots +\beta_{n}X_{n})]}\in [0:1]$$ (3)

The general expression of the logistic regression

$${Y=logit(p)=\ln(\frac{p}{1-p})=\beta_{0}}_{}+\beta_{1}X_{1}+\beta_{2}X_{2}+\ldots +\beta_{n}X_{n}+\varepsilon$$ (4)

Where: *p* is the likelihood of banana presence; β_0_, β_1_, β_2_, …, β_n_ are coefficients of the factors influencing banana spatial distribution; *X*_1_, *X*_2_, …, *X*_n_ are factors influencing banana distribution; *z* are the linear combinations of β_*i*_ and *X*_*i*_; *Y* is the response variable with values of *p* ∈ [0:1]; ε is the error term (mean ≠0 and variance dependent on *X*_*i*_).

Our training dataset with 12 covariates was used for fitting logistic regression models representing different complexities: the null model without covariates (M0-12); the full model with all covariates (M1-12); expansion of M1-12 that includes significant two-way interactions (M2-12). All terms that would not maximise predictive power of M2-12 were iteratively excluded via stepwise regression implemented with the stepAIC function of the R package MASS [66]. Model goodness-of-fit was determined based on the smallest values of log-likelihood, deviance, Akaike Information Criterion (AIC), and Bayesian Information Criterion (BIC) [67]. The best performing model was used to identify covariates that best explained the variance in the presence/absence of banana. We also made predictions using the testing dataset to evaluate the performance of our logistic regression models using the metric ROC AUC. Prediction maps of banana distribution were generated through indicator regression kriging [68] while the presented figures were created using QGIS [23].

### Geographic shifts and the associated biophysical factors

Analysing geographic shifts in the occurrence of banana and potential underlying factors can help different stakeholders along the banana value chain to design strategies to increase productivity and enhance sustainability for the future. In this study, the years 1958 and 2016 were the basis for analysing spatial changes in the distribution of banana in Uganda over a period of 50 years. First, the current (2016) map of banana distribution was resampled from 250 m to 5 km resolution, matching the gridded map of historical (1958) banana distribution. From our assessment, we noticed that if banana presence was due to a single banana field, then it would be much easier to find it in a 250 m × 250 m pixel compared to a 5 km × 5km pixel. The regional share of banana in both 1958 and 2016 were computed using counts of pixels with banana in each region divided by the total number of pixels with banana in Uganda. Second, the gridded maps were overlaid to determine the areas where banana cultivation has decreased (-1), increased (1) and remained stable (2). Third, the geographic shift map was overlaid using spatial polygons of administrative regions, and agroecological zones (S1 Fig) [16]. This facilitated computation of the percentage of the geographic shift in each region and agroecological zone, determined by dividing the number of pixels corresponding to the different shift categories by the total number of pixels of the geographic shift map of Uganda. Fourth, classification and regression trees (CART), a nonparametric method that recursively splits distribution categories in terms of the extracted values of the covariate variables [56] was performed to identify key biophysical factors that could have contributed to geographic shift in banana. Data used for CART comprised of geographic shift categories and significant covariates of the best performing logistic regression model. We performed CART using the rpart model using the rpart function of the R package rpart because it keeps track of the complexity of the tree (i.e., size of the tree) and separates the classes of the target variable [69]. Model fitting used geographic shift categories for the dependent variable and normalised covariates as the explanatory variables. Visualization of rpart model outputs was done using the rpart plot [70].

## Results

### Effect of spatial filtering on point pattern distribution

Spatial filtering resulted in the retention of 7,632 unique data points (1,518 presence; 6,114 absence). Average nearest neighbour analysis implemented with the unfiltered 18,949 data points revealed a clustered point pattern with less than 1% likelihood of being due to random chance (Table 3). However, the change in the *z*-score from -56.117 to 0.056 indicates that spatial filtering helped to achieve the random point pattern required to ensure that extracted covariates satisfy the assumptions of spatial independence (Table 3).

**Table 3:** Average nearest neighbour analysis before and after filtering of data points.

Average nearest neighbour	Before	After
	(*n* = 18,949)	(*n* = 7,632)
Observed mean distance	1725.313	3453.793
Expected mean distance	2192.531	3452.629
Nearest neighbour ratio	0.786905	1.000337
z-score	-56.11747	0.056336
p value	0.000000	0.955074
Type of point pattern	Clustered	Random

### Algorithm performance under different subsampling scenarios

**Fig 4:**
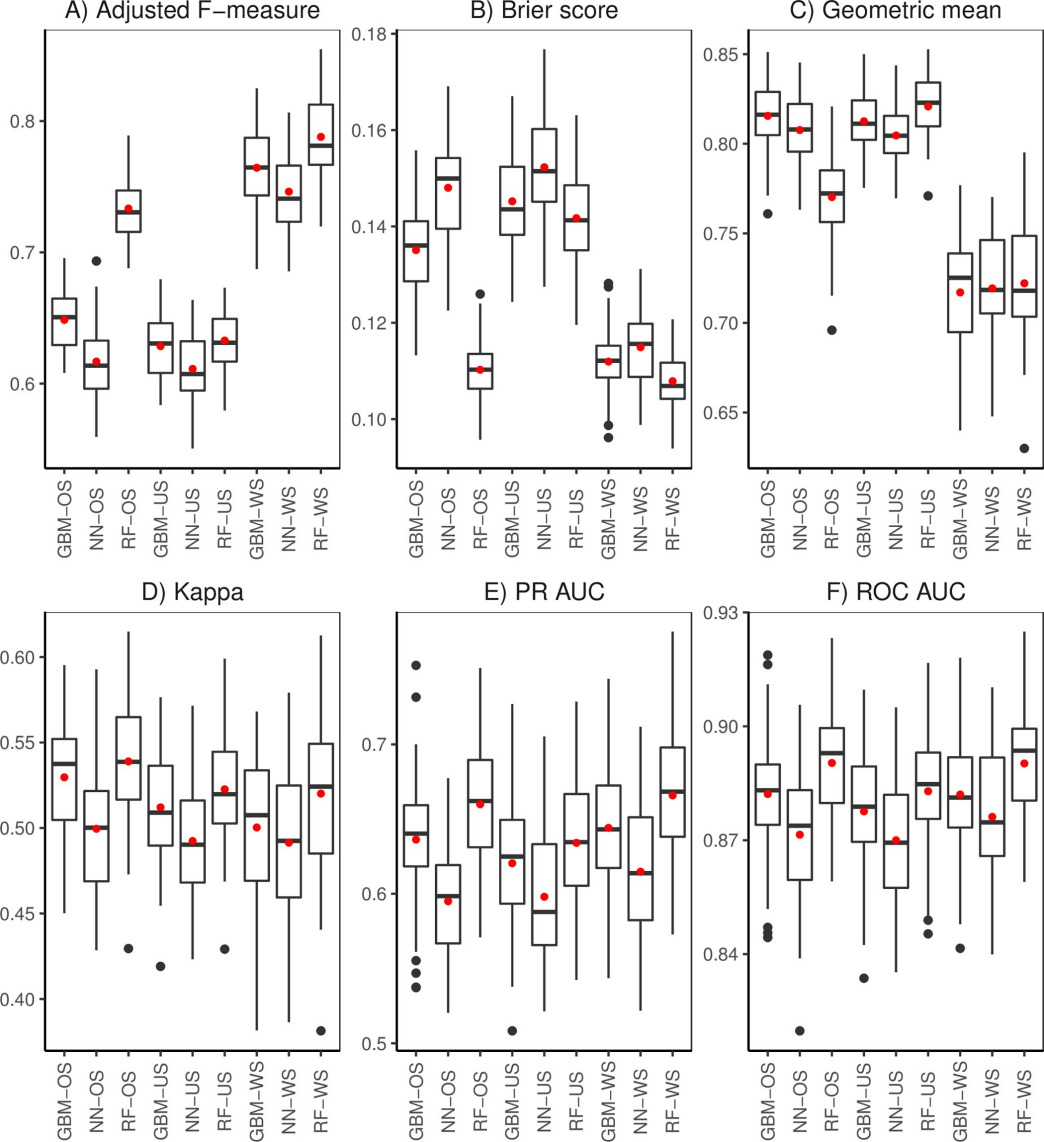
Performance metrics (A: Adjusted F-measure, B: Brier score, C: Geometric mean, D: Cohen’s Kappa, E: PR AUC, F: ROC AUC) for random forest (RF), gradient boosted machines (GBM) and neural networks (NN) trained on the 12 covariates chosen via subjective feature selection. Each algorithm was trained under three different sampling scenarios: Oversampling (OS), and undersampling (US) and without sampling (WS). The black line and red dot inside the box are the median and mean, respectively.

**Fig 5:**
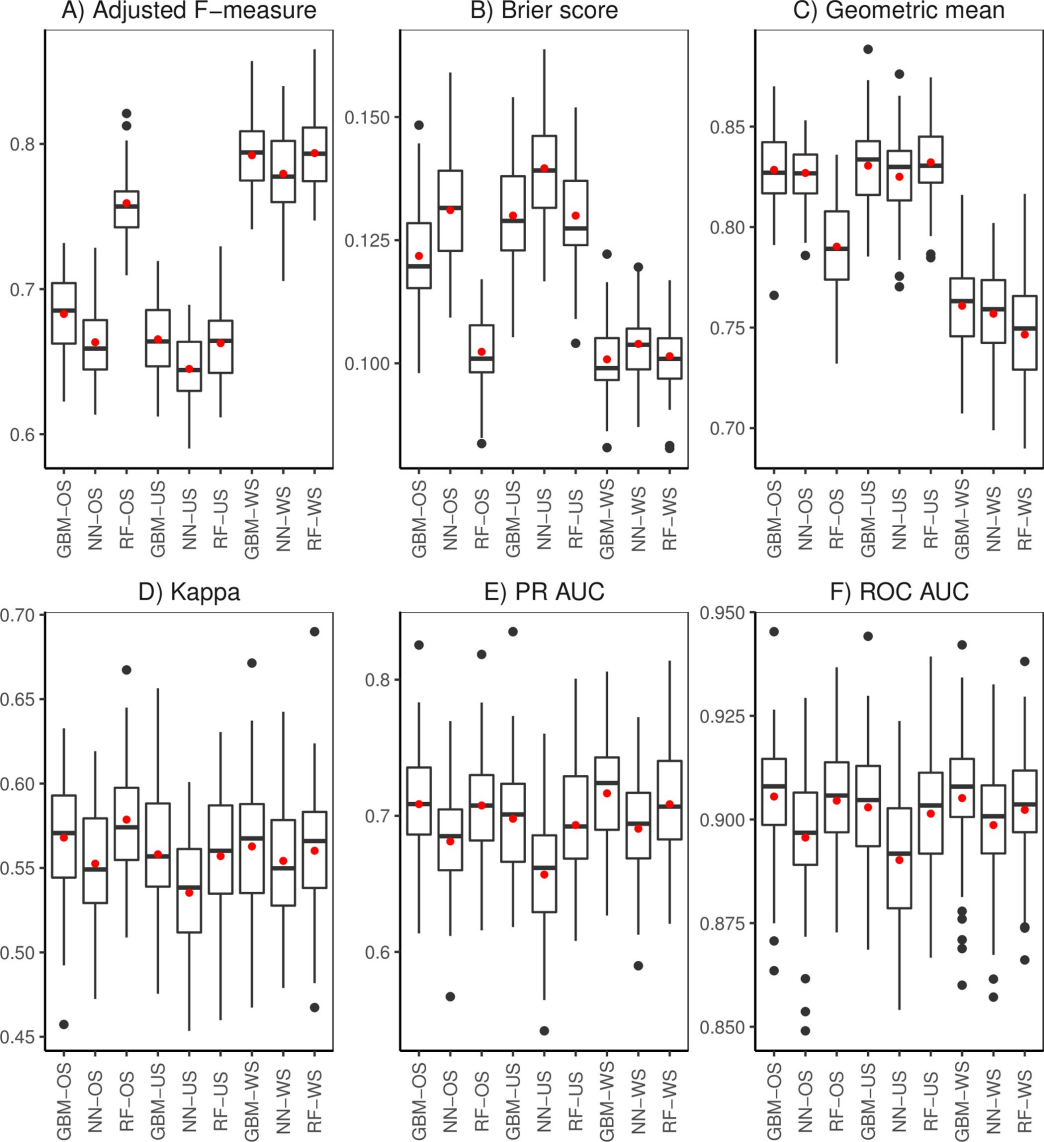
Performance metrics (A: Adjusted F-measure, B: Brier score, C: Geometric mean, D: Cohen’s Kappa, E: PR AUC, F: ROC AUC) for random forest (RF), gradient boosted machines (GBM) and neural networks (NN) trained on the 17 covariates selected using recursive feature elimination. Each algorithm was trained under three different sampling scenarios: Oversampling (OS), undersampling (US) and without sampling (WS). The black line and red dot inside the box are the median and mean, respectively.

Resampling of the training data helps to resolve the disparity in the frequencies of presence/absence observations (class imbalance) which can have significant negative impact on model fitting and subsequent predictions. We examined the performance of RF, GBM, and NN trained on the 12 and 17 selected covariates under diverse resampling scenarios (Figs 4; 5). Effects of oversampling (OS) (i.e., adjusted F-measure, Brier score, Geometric mean, PR AUC, and ROC AUC) suggest that it significantly affects the performance across algorithms. RF-OS outperformed GBM-OS and NN-OS for all metrics except for the Geometric mean and Kappa. Results for undersampling (US) show no significant difference between RF and GBM evaluated using all metrics apart from the Geometric mean. We found that NN-US consistently registers significantly poor results with all evaluation metrics (Fig 4A–4F). Without sampling (WS) did not cause a significant difference among algorithms as regards the Geometric mean. However, it did significantly influence algorithms when measured using the adjusted F-measure and PR AUC. RF had significantly better average values of adjusted F-measure, Brier score, Kappa, PR AUC, ROC AUC (Fig 4A–4F). Similarities in the results presented in Figs 4 and 5 relate to oversampling (i.e., adjusted F-measure, Briers score and Kappa) and undersampling (i.e., adjusted F-measure, Briers score and Kappa, PR AUC, and ROC AUC). Algorithms performed significantly better without resampling (WS) with respect to the adjusted F-measure and Brier score. All algorithms trained had similar average values of Kappa, PR AUC, and ROC AUC for the scenarios examined. The significantly lower Geometric mean for algorithms without sampling indicates that doing nothing to resolve the problem of class imbalance has a negative impact on performance. In general, OS results in fluctuations in performance among algorithms compared to US which provides more consistent results among algorithms. This demonstrated the appropriateness for US to resolve class imbalances in our spatial data. Prediction maps generated from training the algorithms on 12 and 17 covariates without sampling, oversampling and undersampling are available in S2 and S3 Figs, respectively.

### Comparison of ensemble models

**Fig 6:**
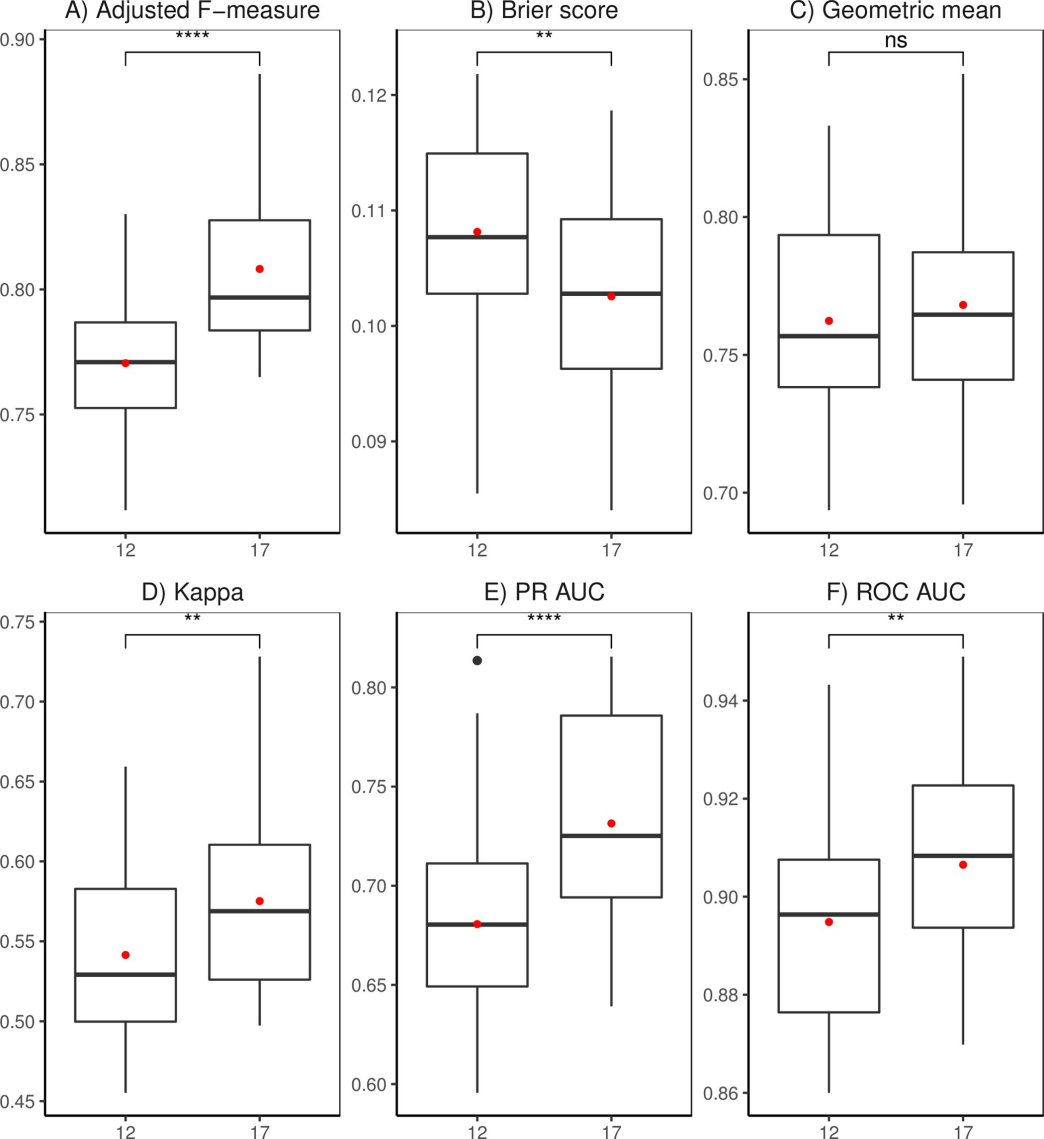
Performance metrics (A: Adjusted F-measure, B: Brier score, C: Geometric mean, D: Cohen’s Kappa, E: PR AUC, F: ROC AUC) for the ensemble models. The black line and red dot inside the box are the median and mean, respectively. Wilcoxon rank test significance values: Not significant (ns) p > 0.05; * p < 0.05; ** p < 0.01; *** p < 0.001; ****p < 0.000.

**Fig 7:**
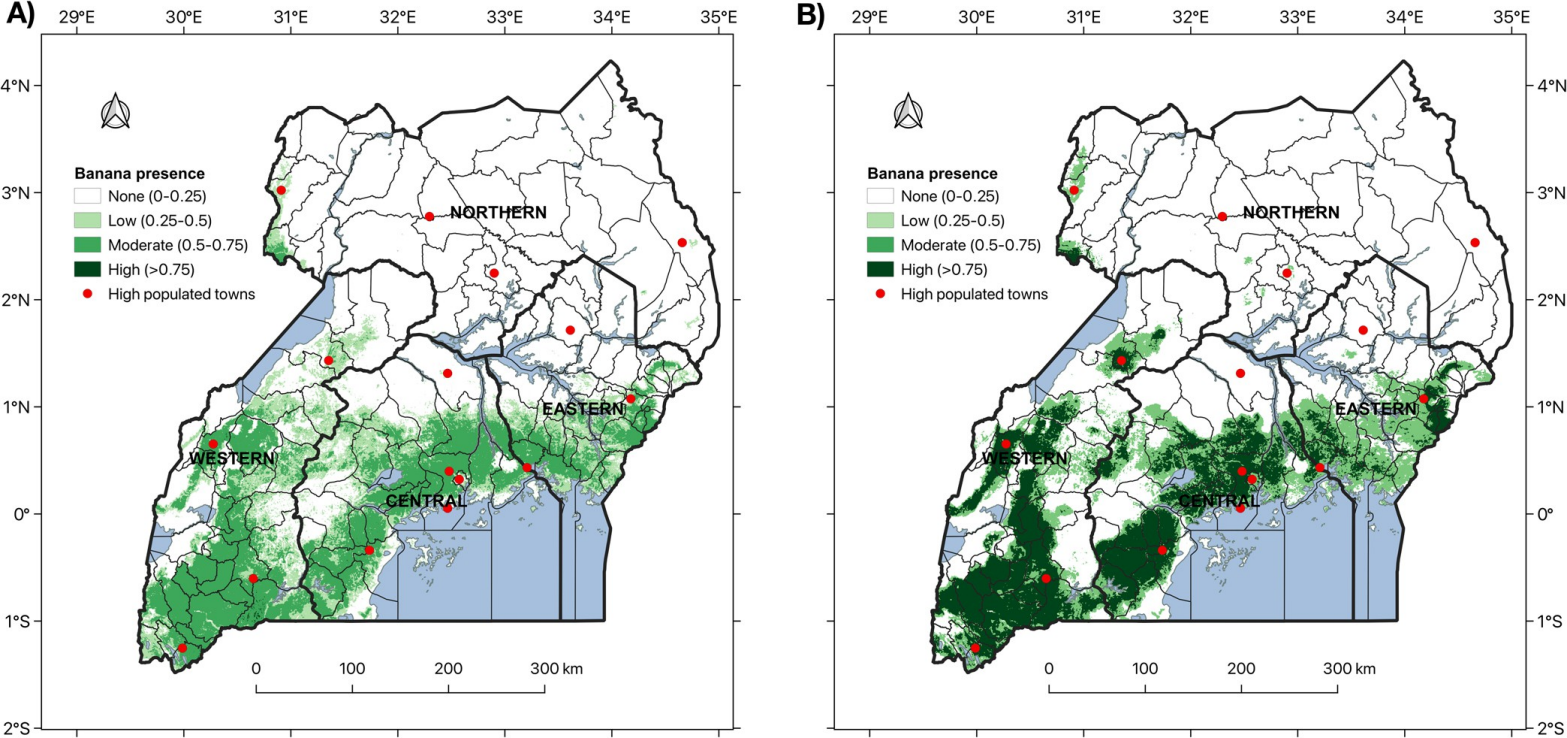
Predicted banana distribution map (2016) using an ensemble model from RF, GBM and NN trained on A) 12 covariates and B) 17 covariates. The maps were refined using the SAGA majority filtering tool within QGIS. Probabilities were converted into categories of banana presence using the probability threshold of 0.25 that maximizes the true positive rate and true negative rate (Max TPR+TNR).

Fig 6 shows that the number of covariates on which the algorithms were trained has significant influence on the performance of the built ensemble model. The ensemble from algorithms trained on the 17 covariates had significantly better performance with respect to the Adjusted F-measure, Brier score, Kappa, PR AUC, and ROC AUC (Fig 6A, 6B; 6D; 6E and 6F). Recursive feature selection of the 17 covariates enabled the unbiased selection of 2 socio-economic and 15 biophysical variables that best explained the observed banana presence/absence variation. In contrast, Geometric mean reveal no significant difference between ensembles from algorithms trained on 12 and 17 covariates (Fig 6C). Our predicted spatial coverage of banana demonstrates that the ensemble of algorithms trained on 12 covariates highlights locations of low and moderate presence probabilities, and few patches of high presence probability particularly within Isingiro district in western Uganda (Fig 7A). In contrast, the ensemble of algorithms trained on 17 covariates emphasises the presence locations with a high probability (Fig 7B). Both ensemble prediction maps reveal that banana is scarce in the northern region but common in a band that extends from the western through central into the eastern region (Fig 7A and 7B). The foothills of Mount Ruwenzori, Southwestern Highlands, northern and western shores of Lake Victoria and the foothills of Mount Elgon standout as the hotspots of banana production (Fig 7A and 7B). Additionally, there is some correlation between the accentuated banana zones with a high presence probability and densely populated cities and towns (Fig 7B). The socio-economic variables (population density and travel time to a market of 500,000 people) represent better market prospects for crop products and improved access to inputs and services due to the physical proximity to populated cities and towns [71]. Only five of the biophysical variables appeared to have a direct relationship with presence or absence of banana. Therefore, logistic regression model fitting was restricted to the 12 covariates connected with banana growth chosen via subjective feature selection.

### Performance of the fitted logistic regression models

The goodness-of-fit statistics indicate that the M2-12 model with significant two-way interactions between covariates included had better performance with respect to the log-likelihood, deviance, AIC, BIC, and ROC AUC (Table 4). We found that adding significant three-way interactions to M2-12 increased the likelihood and ROC AUC, but the model would suffer a penalty on the BIC (results not included). M2-12 was thus selected as the most parsimonious and interpretable model for explaining the banana distribution.

**Table 4:** Comparison between logistic regression models of different complexity and structure derived from the 12 covariates chosen using subjective feature selection.

Model^a^	DF	Model fit statistics ^c^					Analysis of deviance ^b^		
		Log-like	Deviance	AIC	BIC	ROC AUC	M0-12	M1-12	M2-12
M0-12	1	-2392.6	4785.2	4787.2	4793.6	0.5			
M1-12	13	-1751.5	3503.0	3529.0	3612.3	0.830	1282.1		
							***		
M2-12	46	**-1501.0**	**3001.9**	**3093.9**	**3388.7**	**0.879**	1783.2	501.1	
							***	***	

^a^ M0-12: glm(y~1), the null model; M1-12: glm(y~(x_1_+x_2_+…+x_n_)), full model with 12 covariates; M2-12: glm(y~(x_1_+x_2_+…+x_n_)^2), full model including also significant two-way interactions; x and y represent the independent and dependent variables, respectively; n is the number of covariates in the model. DF, degree of freedom.

^b^ Chi-square *X*^2^) values followed by *** are significantly different at p ≤ 0.000.

^c^ Log-Like, Log likelihood, Deviance, Residual deviance, AIC, Akaike Information Criteria, BIC, Bayesian Information Criteria, ROC AUC, Receiver Operator Characteristic Area Under Curve.

### Key factors explaining banana distribution

The estimates obtained from fitting the model M2-12 reveals a significant positive influence of BD, RB3BLUE, PH, PREC, SLOPE and SOC and a significant negative influence of AMT and PSEA (Table 5). Significant interactions of certain covariates that appear to have no significant influence demonstrates the cross-over existing between covariates in the real world (Table 5).

**Table 5:** Summary results of the logistic regression model M2-12 including the significant two-way interactions to maximise loglikelihood.

Variable ^a^	Estimate	Standard error	Pr(>|z|) ^b^	Signif. ^c^
(Intercept)	-1.54678	0.13822	0.00000	***
AMT	-1.88570	0.20330	0.00000	***
ATR	0.17487	0.15941	0.27264	ns
BD	0.32422	0.11068	0.00340	**
RB3BLUE	0.87111	0.27957	0.00183	**
CEC	-0.12797	0.18768	0.49535	ns
FAPARvar	-0.12381	0.10350	0.23159	ns
PH	0.39193	0.18416	0.03332	*
PREC	0.92522	0.14596	0.00000	***
PSEA	-1.23541	0.14284	0.00000	***
SAND	0.01594	0.12305	0.89692	ns
SLOPE	0.30466	0.09359	0.00113	**
SOC	0.57015	0.17837	0.00139	**
AMT:BD	-0.49688	0.11945	0.00003	***
AMT:RB3BLUE	1.10388	0.19493	0.00000	***
AMT:CEC	0.37685	0.17479	0.03108	*
AMT:PH	-0.99216	0.19456	0.00000	***
AMT:PSEA	0.32788	0.18623	0.07831	ns
ATR:RB3BLUE	-0.36877	0.24932	0.13912	ns
ATR:PH	1.08080	0.18943	0.00000	***
ATR:PREC	0.40241	0.13002	0.00197	**
ATR:PSEA	-0.50433	0.13405	0.00017	***
ATR:SAND	0.29075	0.10360	0.00501	**
ATR:SOC	0.24250	0.14119	0.08587	ns
BD:CEC	-0.41079	0.11244	0.00026	***
BD:PH	0.38239	0.15333	0.01264	*
BD:PSEA	0.18451	0.09654	0.05598	ns
BD:SAND	-0.15018	0.08712	0.08475	ns
BD:SOC	-0.21739	0.11208	0.05244	ns
RB3BLUE:CEC	0.85900	0.26187	0.00104	**
RB3BLUE:FAPARvar	0.40279	0.14081	0.00423	**
RB3BLUE:PH	-1.47099	0.27023	0.00000	***
RB3BLUE:PSEA	0.66855	0.19178	0.00049	***
RB3BLUE:SAND	-0.39036	0.12896	0.00247	**
CEC:PH	-0.47655	0.14682	0.00117	**
CEC:PSEA	-0.28238	0.16449	0.08604	ns
CEC:SAND	-0.23913	0.15338	0.11899	ns
CEC:SLOPE	-0.41289	0.11396	0.00029	***
CEC:SOC	-0.34385	0.17305	0.04693	*
PH:SOC	0.29831	0.18076	0.09888	ns
PREC:PSEA	0.31757	0.12801	0.01311	*
PREC:SAND	-0.15627	0.09509	0.10030	ns
PREC:SLOPE	-0.11390	0.06906	0.09908	ns
PREC:SOC	-0.38969	0.12277	0.00150	**
PSEA:SOC	0.37027	0.14591	0.01116	*
SAND:SOC	-0.20258	0.10779	0.06019	ns

^a^ Table 2 for the full names and units of the variables. The sign ‘:’ indicates the interaction between terms.

^b^ Pr(>|z|), probability of the *z*-score.

^c^ Significance: *** *p* < 0.001; ** *p* < 0.01; * *p* < 0.05; not significant (ns) *p* > 0.05

**Fig 8:**
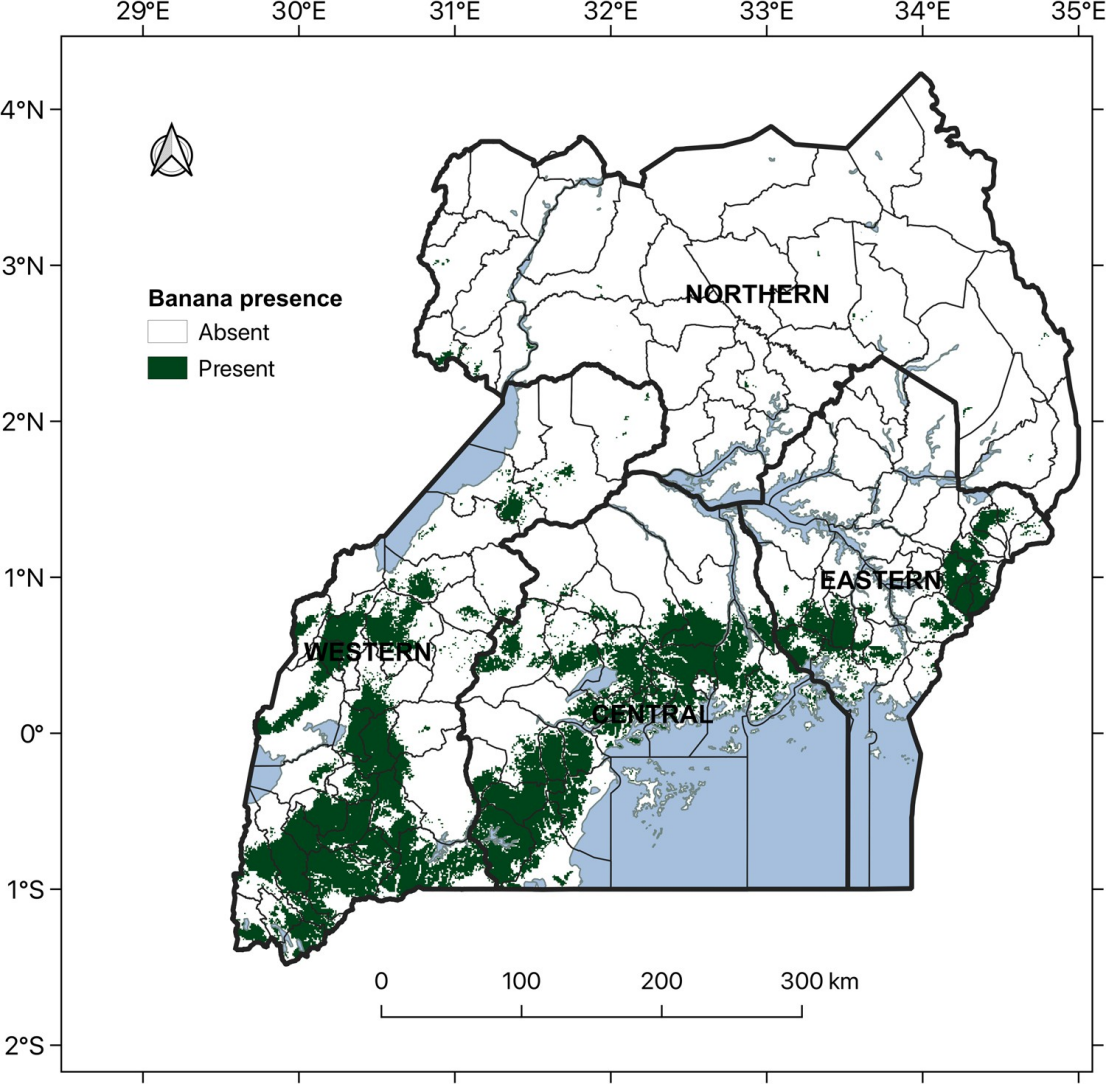
Predicted banana distribution map (2016) using logistic regression model M2-12 fitted using 12 covariates and significant two-way combinations.

Fig 8 shows the latest banana distribution map created using indicator regression kriging [72] with prediction outputs of the logistic regression model M2-12. This was selected because its predictor variables had a direct role in banana growth unlike in the ensemble model and yet its performance indices were similar to those for the ensemble model with 12 covariates. The map highlights active banana production on the northern and western shores of Lake Victoria, the Southwestern highlands, the foothills of Mount Ruwenzori and of Mount Elgon (Fig 8). This is consistent with what was revealed by the ensemble prediction maps.

### Spatial coverage and geographic shifts of banana

**Fig 9:**
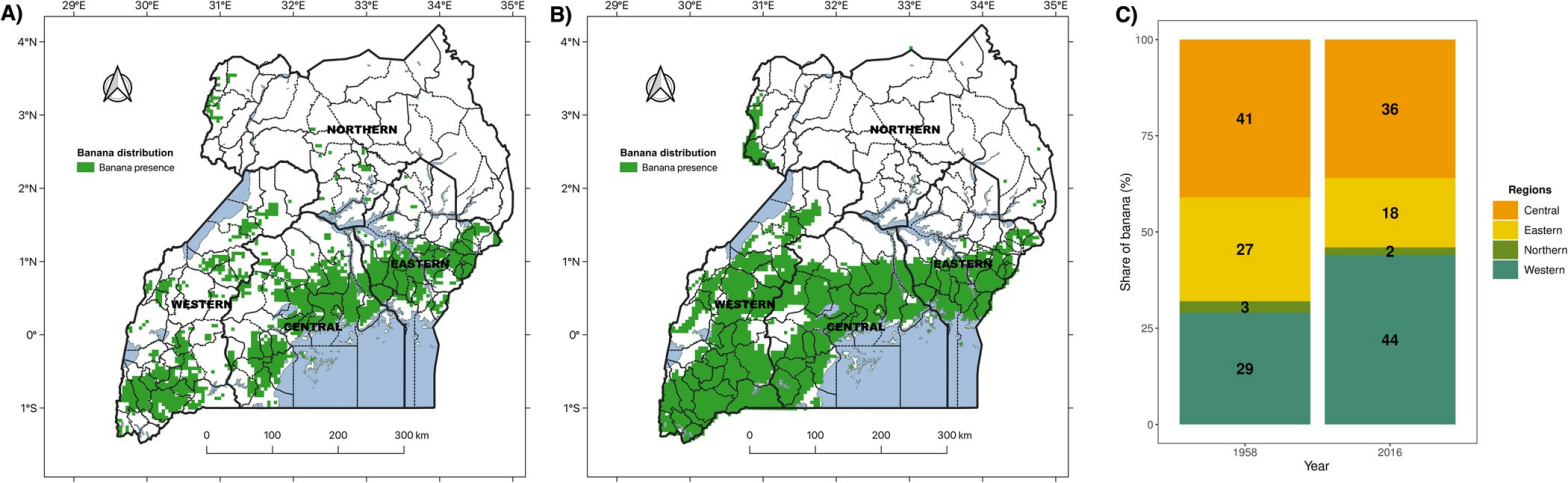
Spatial distribution of banana A) historical banana distribution (1958); B) latest banana distribution (2016) predicted using ensemble model of RF, GBM and NN trained on the 12 covariates; C) percentage share of banana among administrative regions: Northern, Eastern, Central and Western. The share of banana was computed using counts of pixels with banana in each region divided by the total number of pixels with banana in Uganda.

In 1958, most banana was grown in the central (41%), western (29%) and eastern (27%) regions of Uganda (Fig 9A and 9C). The current (2016) distribution of banana reveals that the western (44%) had overtaken the central (36%) as the region with the largest banana coverage of the cropping system in Uganda (Fig 9B and 9C). Banana coverage has remained sparse in the northern region (Fig 9A, 9B and 9C).

**Fig 10:**
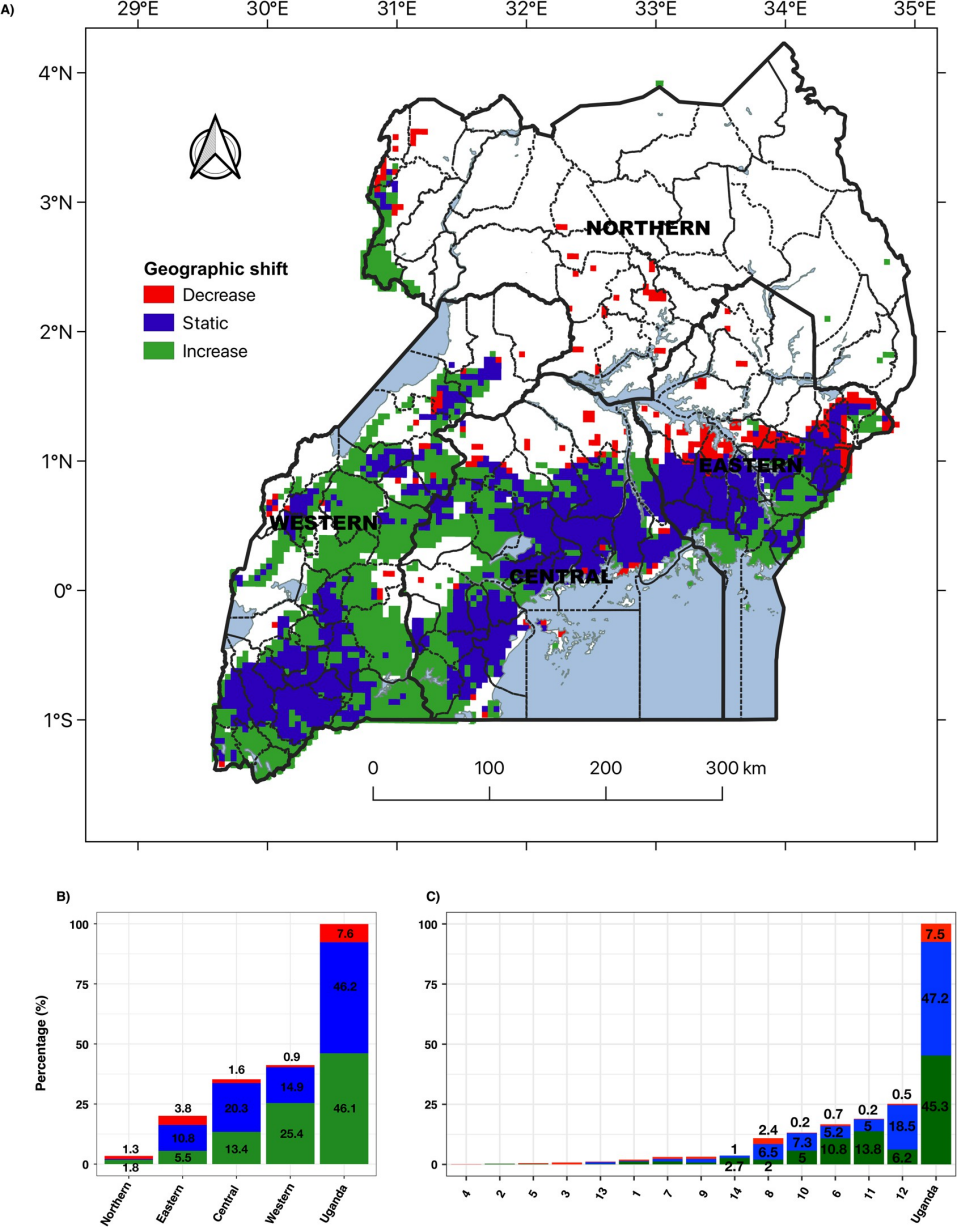
Geographic shifts of banana in Uganda. A) geographic shift patterns generated by overlaying the historical distributions (1958) and latest banana distribution (2016); B) percentage distribution of banana geographic shift between administrative regions: Northern, Eastern, Central and Western; C) percentage distribution of banana geographic shift among agroecological zones 1: West Nile Farmlands; 2: Northwestern Farmlands-Wooded-Savanna; 3: Northern Moist Farmlands; 4: Northeastern Central Grass-Bush Farmlands; 5: Northeastern Semi-arid Short Grass Plains; 6: Western Mid-Altitude Farmlands and the Semuliki Flats; 7:Central Wooded Savanna; 8: Southern and Eastern Lake Kyoga Plains; 9: Mount Elgon Farmlands; 10: Western Medium High Farmlands; 11: Southwestern Grass Farmlands; 12: Lake Victoria Crescent and Mbale Farmlands; 13: Ssese Islands and Sango Plains; 14: Southwestern Highlands. The percentages were computed based on numbers of pixels in each region that correspond to the different shift categories divided by the total number of pixels in the geographic shift map of Uganda.

Geographic shifts of banana were classified by locations where cultivation has decreased (8%), increased (46%), or remained stable (46%) (Fig 10A). At the national level, about 60% of the increased banana cultivation was in the western region (Fig 10B). The central region contained 44% of the banana cultivation areas that remained stable between 1958 and 2016 (Fig 10B). Half (50%) of the national decrease in banana cultivation occurred in the eastern region (Fig 10B). There is a comparable increase and decrease in scattered banana locations in the northern region (Fig 10B). At the agroecological zone level, a geographic shift is significant in five of the fourteen agroecological zones of Uganda: Southern and Eastern Lake Kyoga Plains, Western Medium High Farmlands, Western Mid-Altitude Farmlands and the Semuliki Flats, Southwestern Grass Farmlands and Lake Victoria Crescent and Mbale Farmlands (Fig 10C). What stands out is that the Lake Victoria Crescent and Mbale Farmlands accounts for about 40% of the stable cultivation at the national level, Southern and Eastern Lake Kyoga Plains for about 32% of the decreased cultivation, and Southwestern Grass Farmlands for 30% of the increased cultivation (Fig 10C).

### Biophysical factors associated with geographic shifts of banana

**Fig 11:**
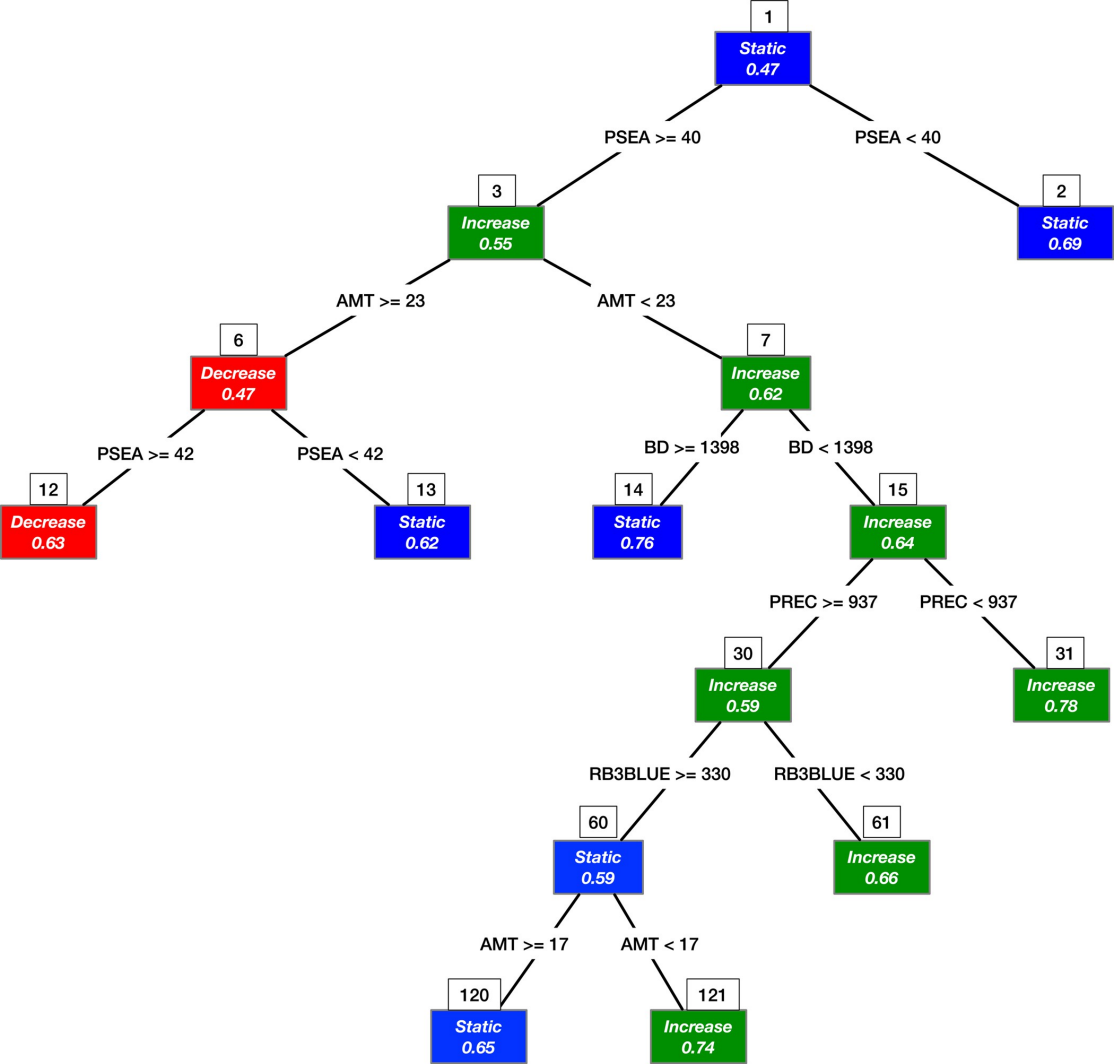
Classification and regression tree (CART) showing the biophysical factors associated with geographic shift in banana at national level. Probabilities for each geographic shift class are included within the coloured boxes. The node number at which a split occurs are shown above the coloured boxes.

We performed the CART analysis using the geographic shift classes and the eight significant covariates of the model M2-12 (BD, RB3BLUE, PH, PREC, SLOPE, SOC, AMT, and PSEA) for the dependent variable and explanatory variables, respectively. CART selected PSEA, AMT, BD, PREC, and RB3BLUE for tree construction (Fig 11). The root node of the tree represents areas where banana cultivation remained stable. The criteria PSEA < 40% splits the root node into the left and right-hand branches if true and false, respectively (Fig 11). The left-hand branch shows a probability of 69% that banana cultivation remained stable in areas with mild dry periods (PSEA < 40%), characteristic of the Lake Victoria Crescent and Mbale Farmlands and Western Medium High Farmlands. In contrast, the right-hand branch indicates a probability of 55% that banana cultivation increased in areas that experienced severe dry periods (PSEA ≥ 40%). A probability of 63% at terminal node 12 reveals that banana cultivation decreased in areas where high temperature (AMT ≥ 23°C) worsened the negative effects of severe dry periods (PSEA ≥ 42%). On the contrary, terminal node 13 indicates a 62% probability of stable cultivation in locations where mild dry periods (PSEA < 42%) reduced the negative effects of high temperature (AMT ≥ 23°C). Similarly, the right-side split of node 3 reveals a 62% probability that banana cultivation increased in locations where lower temperatures (AMT < 23°C) offset the negative effects of severe dry periods (PSEA ≥ 40%) (Fig 11). The terminal node 14 associates 76% probability of stable cultivation with PSEA ≥ 40%, AMT < 23°C, and BD ≥ 1398 kg m^-3^. Additionally, a series of right-side splits that terminate at node 31 show significant increase in the probability of increased cultivation from 62% to 78% when PSEA ≥ 40%, AMT < 23°C, BD < 1398 kg m^-3^ and PREC < 938 mm. The left-side split at node 15 coupled with a right-side split at node 30 that terminated at node shows a 66% probability of increased cultivation. Moreover, the left-side split at node 30 that terminated at nodes 120 and 121 indicates a 65% and 74% probability of stable and increased cultivation, respectively. The above evidence intimates that factors associated with crop water requirements have facilitated geographic shift (or lack of it) in the banana cropping system over the past five decades.

## Discussion

We mapped the distribution of banana by collecting presence/absence data from high-resolution satellite imagery. Coupling the presence/absence information with multiple gridded covariates resulted in a robust geospatial dataset for predicting the spatial distribution of banana. Performing data pre-processing was prudent to deal with general issues often associated with large spatial datasets for prediction modelling. Multicollinearity detected among the acquired 71 covariates underscored the need to select relevant and independent covariates so that built models are not subject to overfitting. Other issues we identified were spatial autocorrelation and class imbalance of response variables. These two problems are often ignored in spatial prediction modelling, yet they can increase spatial bias and uncertainty especially when model predictions must be made outside the scope of learned relationships between covariates and response outcomes [73].

Our second objective was to identify approaches for predicting the spatial distribution of banana using remote sensing data. Spatial filtering helped to ensure randomness of the outcome but could not eliminate class imbalance and residual spatial autocorrelation. We accounted for class imbalance and residual autocorrelation by resampling. The effects of oversampling and undersampling on the accuracy of random forests (RF), gradient boosting machines (GBM) and neural networks (NN) were compared. Unlike oversampling which caused fluctuations in performance between the algorithms, the results from undersampling were more consistent which favoured its selection to resolve class imbalance. Robinson et al. [74] found that undersampling increased the accuracy of machine learning models when both spatial filtering and resampling are used to correct for class imbalance and spatial bias. Compared with the algorithms trained on 12 and 17 covariates with undersampling, the performance of the ensemble models was superior based on the metrics Adjusted F-measure, Brier score, Kappa and PR AUC and ROC AUC. Others have documented the robustness and accuracy of ensemble models [75]. This is in line with various studies of ecosystem services that have endorsed ensemble models as a cost-effective decision support tool in formulating evidence-based policies and actions in sustainable development [76]. Yet, the logistic regression (ROC AUC = 0.879) performed nearly as well as the ensemble models (ROC AUC = 0.895; 0.907 with 12 and 17 covariates, respectively), as others have observed [77].

The third objective focused on determining biophysical covariates that influenced the spatial distribution of banana. Logistic regression facilitated making inferential statements about the relationship the 12 subjectively chosen covariates and the presence or absence of banana. Model estimates revealed a positive influence of blue reflectance, soil organic carbon annual precipitation, slope gradient, soil pH and soil bulk density, and a negative relationship with mean annual temperature and precipitation seasonality. The reasons associated with crop growth and development that guided subjective selection of 12 covariates helped to rationalize the importance of these covariates. Unlike other covariates that have direct influence on banana growth and development, we incorporated blue reflectance among covariates primarily to correct for atmospheric disturbances due to smoke and cloudiness that occasionally obstructed visual interpretation of satellite images [54]. Banana requires well-drained soils with at least 2 m depth to develop an extensive root system for better water and nutrient uptake [78]. Soil physicochemical factors like bulk density and organic carbon influence growth and development via their effect on water and nutrient availability [51]. Reducing bulk density through tillage can improve soil structure and infiltration with the benefits of enhanced banana root and shoot growth [79]. Banana is very sensitive to water stress and a good yield under rain-fed conditions requires an annual precipitation above 1000 mm evenly distributed over the year. A combination of adequate rainfall and minimum precipitation seasonality explain the banana concentrations at elevations between 900–2000 meters above sea level [80].

For the fourth objective, we assessed geographic shifts in banana production areas using spatially explicit differences between maps of banana distribution in 1958 and 2016. Most bananas were grown in the central region in 1958 but the western region is now the main hub of banana cultivation. Reports on geographic shift of banana have mostly provided details of production timelines, magnitudes of changes, and hypothetical causal factors but limited spatial evidence of the changes [19]. Our results show that banana cultivation has mostly declined in the eastern region, remained stable in the central region, and expanded in the western region. The lifespan of well-managed banana plantations used to exceed 30 years in the central and eastern regions, however, they now last for less than 10 years. Farmers have responded in various ways to the deterioration of their banana plantations by i) replanting within existing fields, ii) establishing new plantations, iii) replacing banana with other crops. The first response is prevalent in the central region, where it is common to encounter gardens integrating banana, coffee, cereals, root crops, and shade trees into an agroforestry system. Increased competition between urban land use and agriculture prevents both expansion of existing plantations and the establishment of new plantations in the central region. A blend of the first and second responses is noticeable in the western region, where market opportunities induced area expansions with marked conversion of native vegetation, rangeland, and other croplands into banana plantations. Most of the banana in the region was grown for domestic consumption but it later evolved into a commercial crop. Existing evidence shows that production declines in the central region provided the impetus for the booming domestic trade witnessed today in the western region. The third response is evident in the eastern region where banana has been replaced with cereals and root crops [20]. The success of coffee on the slopes of Mount Elgon reflects a historical preference for a crop with a higher profit to volume ratio that was relatively easy to transport on foot to markets downhill [81]. The disappearance of banana in some areas has serious implications for food security. Famines are rare where banana is the primary staple crop. The loss of the permanent canopy and self-mulch cover that banana provides can lead to greater soil erosion on hilly landscapes.

In the fifth objective, we used the classification and regression tree (CART) to evaluate the biophysical factors associated with geographic shifts in banana distribution over the past five decades. Our results indicate that the expansion of banana cropping system has largely occurred in either marginal areas with relatively poor soils and low rainfall or in ecologically sensitive areas like the tropical rain forests of western Uganda and erosion-prone steep foot slopes of Mount Ruwenzori. Main drivers of decline in banana cropping system are variables linked to soil water supply. Uganda’s banana cultivation is rain-fed [82] and highly dependent on rainwater capture and retention in the soil for crop uptake. Drought prone areas are found in the Southern and Eastern Lake Kyoga Plains where the soils are derived from lacustrine parent material, which tend to be highly sorted with either fine texture in the valleys or coarse texture on the upslopes. The valleys are generally unsuitable for growing banana due to waterlogging [83]. Meanwhile, the sandy nature of the soils on the upslopes renders them prone to rapid drainage and leaching. It is thus likely that the primary cause of decline in banana coverage in the eastern region was drought stress. Although soil cover with organic mulches helps to conserve moisture there is an urgent need to enhance resilience of banana to climate change induced temperature rises and dry spells [84].

Climatic and edaphic factors alone cannot account for geographic shifts in banana. The role of various biotic and cultural factors cannot be ignored. Banana weevil and nematodes have for several decades contributed to the reduced lifespan of banana plantations in the central and eastern regions. The most damaging and costly diseases are Black Sigatoka, Fusarium wilt and Xanthomonas wilt. Also, transitions in dietary habits of people have influenced the crops cultivated as banana is a staple food in the central, eastern, and western region.

## Conclusions

We set out to map the current distribution of banana in Uganda and to understand how this has changed over the past five decades. The resulting map revealed that banana cultivation was concentrated in the western (44%) and central (36%) regions, with small proportions in the eastern (18%) and northern (2%) regions. A logistic regression model showed that banana distribution was positively influenced by annual precipitation, bulk density, soil organic carbon, soil pH and slope gradient, but negatively influenced by mean annual temperature and precipitation seasonality. Geographic shifts in banana cultivation were defined by areas where the crop has decreased (8%), increased (46%), or remained stable (46%). About 60% of increased cultivation was in the western region, a half of decreased cultivation in the eastern region, and 44% of stable cultivation in the central region. The key variables associated with geographic shifts were biophysical factors related to soil water supply, which signifies the importance of irrigation and soil water conservation to mitigate impacts of climate change induced temperature rises and dry spells. The nature of the geographic shifts emphasises the need to enhance resilience to climate change in the agenda for sustainable intensification of banana.

## Supporting information

S1 Fig Aggregated agroecological zones of Uganda (adapted from Wortmann & Eledu, 1999). Zone 1: West Nile Farmlands; 2: Northwestern Farmlands-Wooded-Savanna; 3: Northern Moist Farmlands; 4: Northeastern Central Grass-Bush Farmlands; 5: Northeastern Semi-arid Short Grass Plains; 6: Western Mid-Altitude Farmlands and the Semiliki Flats; 7:Central Wooded Savanna; 8: Southern and Eastern Lake Kyoga Plains; 9: Mountt Elgon Farmlands; 10: Western Medium High Farmlands; 11: Southwestern Grass Farmlands; 12: Lake Victoria Crescent and Mbale Farmlands; 13: Ssese Islands and Sango Plains; 14: Southwestern Highlands.
(TIF)

S2 Fig Predictions of random forests (RF), gradient boosting machines (GBM), and neural networks (NN) trained on 12 covariates chosen using subjective feature selection. Each algorithm was trained under three different sampling scenarios: Oversampling (OS), undersampling (US) and without sampling (WS). A) RF–OS; B) RF–US; C) RF–WS; D) GBM–OS; E) GBM–US; F) GBM–WS; G) NN–OS; H) NN–US; I) NN–WS.
(TIF)

S3 Fig Predictions of random forests (RF), gradient boosting machines (GBM), and neural networks (NN) trained on 17 covariates selected after recursive feature selection. Each algorithm was trained under three different sampling scenarios: Oversampling (OS), undersampling (US) and without sampling (WS). A) RF–OS; B) RF–US; C) RF–WS; D) GBM–OS; E) GBM–US; F) GBM–WS; G) NN–OS; H) NN–US; I) NN–WS.
(TIF)

S1 Table List of 71 remotely sensed and gridded covariates acquired from the internet. (DOCX)
