# Supplementary figures and images for: Mapping spatial distribution and geographic shifts of East African highland banana (Musa spp.) in Uganda
Source: PLoS One. 2022 Feb 17;17(2):e0263439. doi: 10.1371/journal.pone.0263439 (PMC8853547; doi:10.1371/journal.pone.0263439)

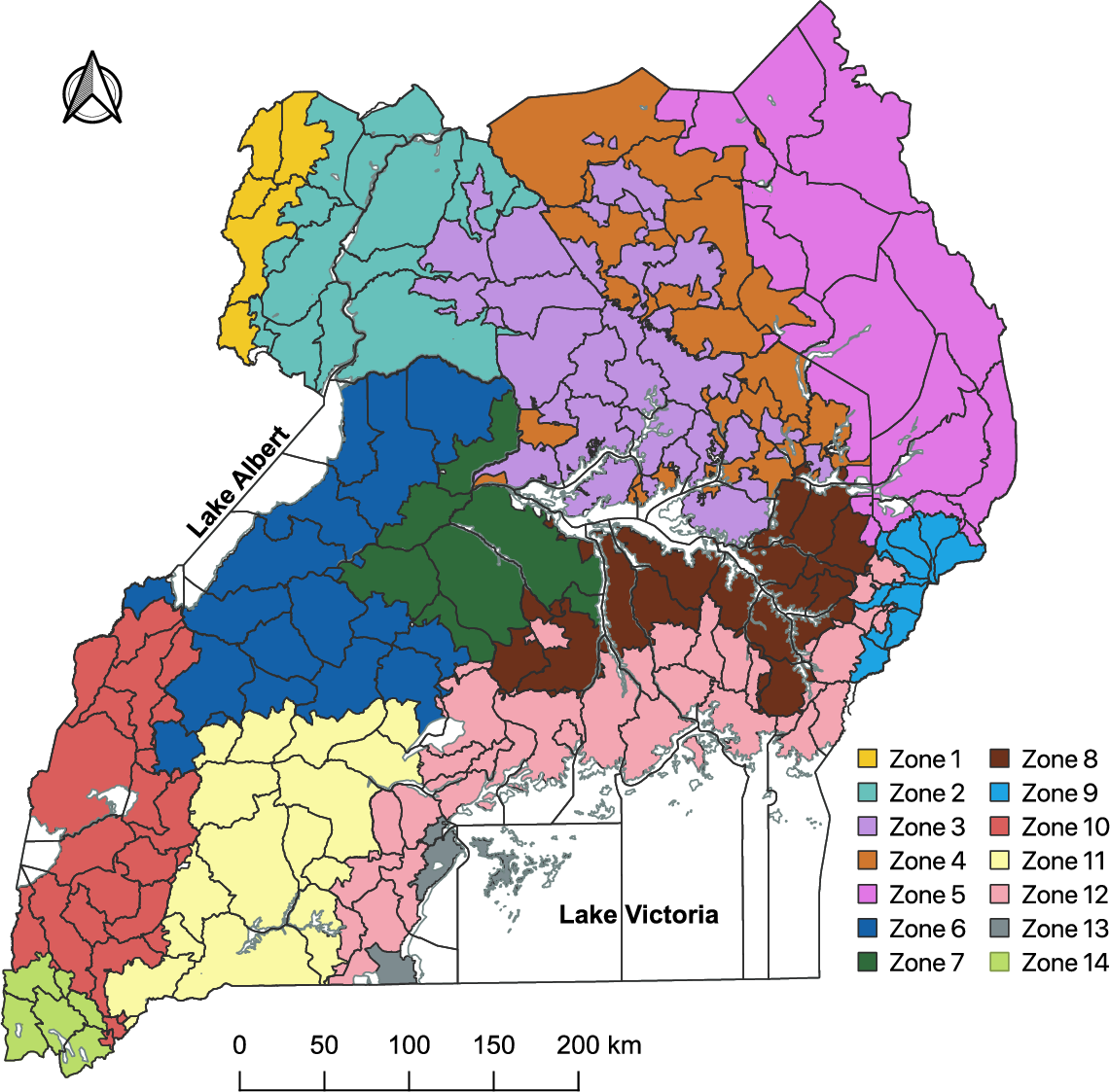

Supplement: S1 Fig — Zone 1: West Nile Farmlands; 2: Northwestern Farmlands-Wooded-Savanna; 3: Northern Moist Farmlands; 4: Northeastern Central Grass-Bush Farmlands; 5: Northeastern Semi-arid Short Grass Plains; 6: Western Mid-Altitude Farmlands and the Semiliki Flats; 7:Central Wooded Savanna; 8: Southern and Eastern Lake Kyoga Plains; 9: Mountt Elgon Farmlands; 10: Western Medium High Farmlands; 11: Southwestern Grass Farmlands; 12: Lake Victoria Crescent and Mbale Farmlands; 13: Ssese Islands and Sango Plains; 14: Southwestern Highlands. (TIF) [file pone.0263439.s001.tif]

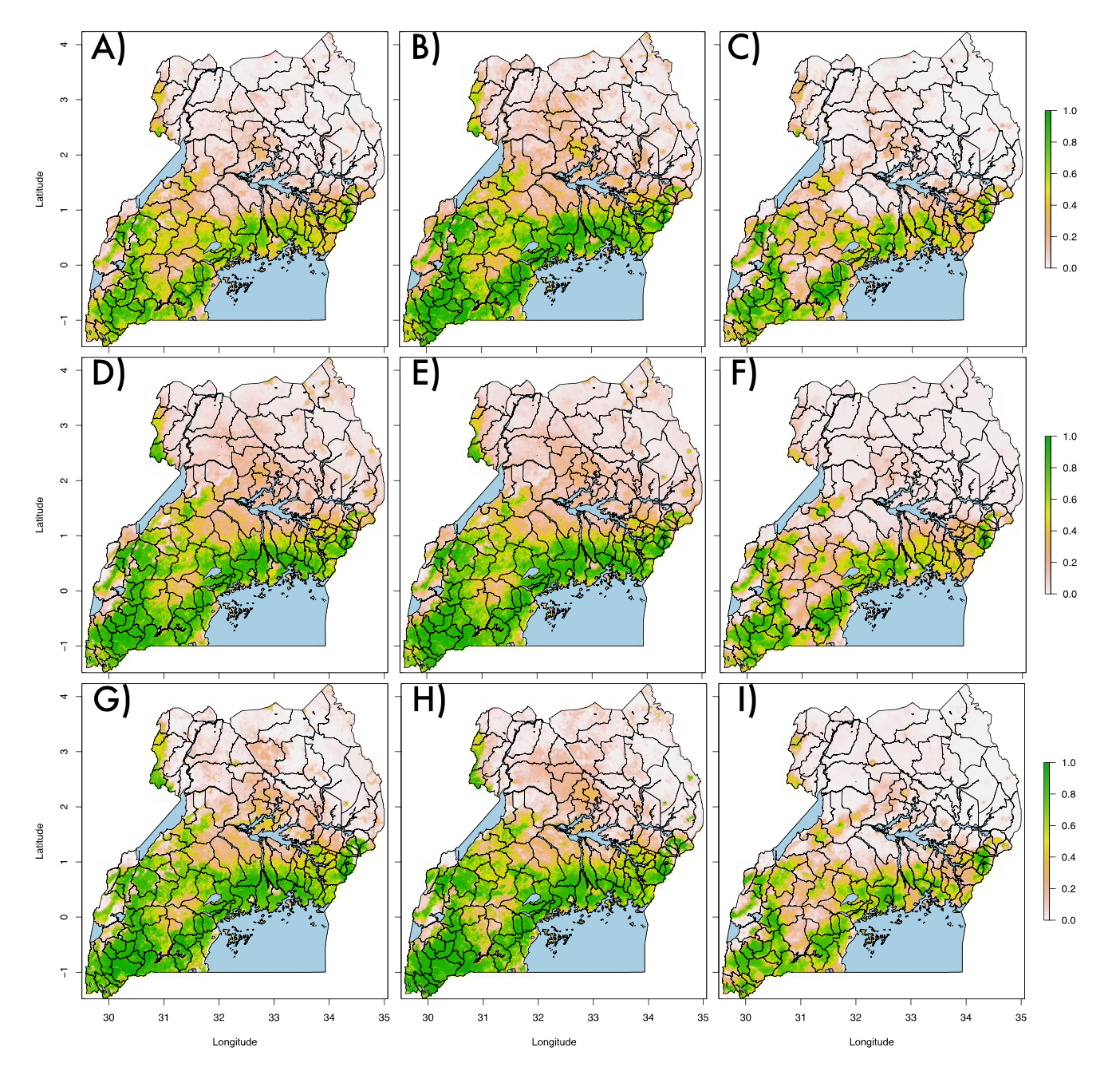

Supplement: S2 Fig — Each algorithm was trained under three different sampling scenarios: Oversampling (OS), undersampling (US) and without sampling (WS). A) RF–OS; B) RF–US; C) RF–WS; D) GBM–OS; E) GBM–US; F) GBM–WS; G) NN–OS; H) NN–US; I) NN–WS. (TIF) [file pone.0263439.s002.tif]

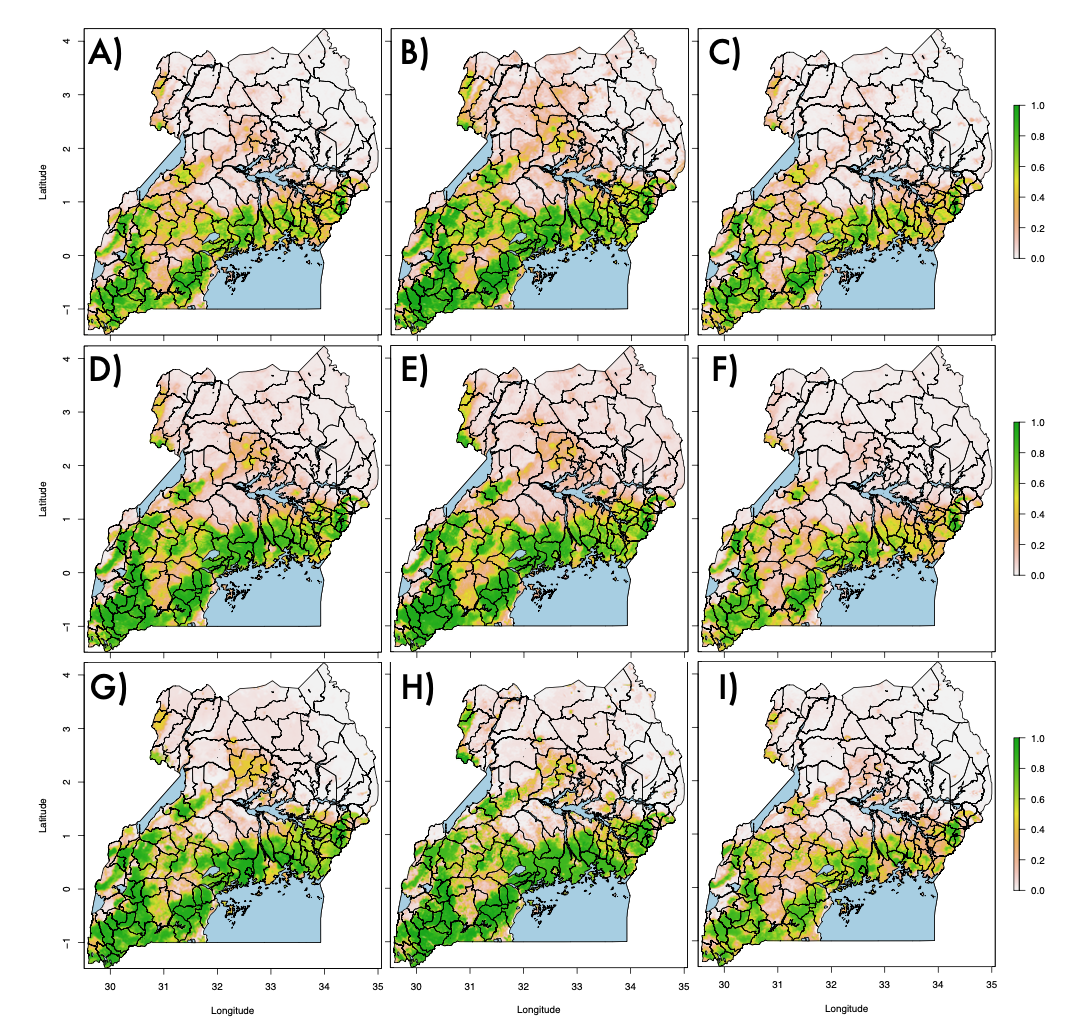

Supplement: S3 Fig — Each algorithm was trained under three different sampling scenarios: Oversampling (OS), undersampling (US) and without sampling (WS). A) RF–OS; B) RF–US; C) RF–WS; D) GBM–OS; E) GBM–US; F) GBM–WS; G) NN–OS; H) NN–US; I) NN–WS. (TIF) [file pone.0263439.s003.tif]
