# Supplementary material for: Mapping spatial distribution and geographic shifts of East African highland banana (Musa spp.) in Uganda
Source: PLoS One. 2022 Feb 17;17(2):e0263439. doi: 10.1371/journal.pone.0263439 (PMC8853547; doi:10.1371/journal.pone.0263439)
Supplement: S1 Table — (DOCX) [file pone.0263439.s004.docx]

**S1 Table. List of 71 remotely sensed and gridded covariates acquired from the internet**

| **Variables** | **Name*** | **Unit** | **Time frame** | **Source** | **Resolution** |
| --- | --- | --- | --- | --- | --- |
| **CLIMATIC FACTORS (n=21)** | | | | | |
| LSTD | Land surface temperature daytime | ^o^C | 2002–2016 | ftp://africagrids.net/1000m/MYD11A2/LST_day/ | 1 km |
| LSTN | Land surface temperature nighttime | ^o^C | 2002–2016 | ftp://africagrids.net/1000m/MYD11A2/LST_night/ | 1 km |
| MFI | Modified fournier index |  | 2002–2016 | <ftp://africagrids.net/1000/> | 1 km |
| CHIRPS | Climate hazards infrared precipitation with stations | mm | 1981–2016 | ftp://africagrids.net/5000m/CHIRPS | 5 km |
| MMAPREC | Mean monthly annual precipitation | mm | 2003-2006 | https://web.archive.org/web/20170621184451/http://www.worldgrids.org/doku.php/wiki:pregsm1 | 1 km |
| MODCLDFOPRED | Cloud forest prediction |  | 2000-2014 | https://data.earthenv.org/cloud/MODCF_CloudForestPrediction.tif | 1 km |
| MODCFINTERSD | Cloud inter-annual variation | SD | 2000-2014 | https://data.earthenv.org/cloud/MODCF_interannualSD.tif | 1 km |
| MODCFINTRASD | Cloud intra-annual variation | SD | 2000-2014 | https://data.earthenv.org/cloud/MODCF_intraannualSD.tif | 1 km |
| MODCFMEAN | Cloud mean annual variation | % | 2000-2014 | https://data.earthenv.org/cloud/MODCF_meanannual.tif | 1 km |
| MODCFSEACONC | Cloud seasonality concentration |  | 2000-2014 | https://data.earthenv.org/cloud/MODCF_seasonality_concentration.tif | 1 km |
| MODCFSEATHTA | Cloud seasonality angle theta |  | 2000-2014 | https://data.earthenv.org/cloud/MODCF_seasonality_theta.tif | 1 km |
| MODCFSEAVISCT | Cloud seasonality single band with color table |  | 2000-2014 | https://data.earthenv.org/cloud/MODCF_seasonality_visct.tif | 1 km |
| MODCFSPATSD1DEG | Cloud variability 1-degree | SD | 2000-2014 | https://data.earthenv.org/cloud/MODCF_spatialSD_1deg.tif | 1 km |
| AMDR | Annual mean diurnal range | ^o^C | 1970-2000 | https://biogeo.ucdavis.edu/data/worldclim/v2.1/base/wc2.1_30s_bio.zip | 1 km |
| AMT | Annual mean temperature | ^o^C | 1970-2000 | https://biogeo.ucdavis.edu/data/worldclim/v2.1/base/wc2.1_30s_bio.zip | 1 km |
| ATR | Annual temperature range | ^o^C | 1970-2000 | https://biogeo.ucdavis.edu/data/worldclim/v2.1/base/wc2.1_30s_bio.zip | 1 km |
| PDrQ | Precipitation of driest quarter | mm | 1970-2000 | https://biogeo.ucdavis.edu/data/worldclim/v2.1/base/wc2.1_30s_bio.zip | 1 km |
| PREC | Annual precipitation | mm | 1970-2000 | https://biogeo.ucdavis.edu/data/worldclim/v2.1/base/wc2.1_30s_bio.zip | 1 km |
| PSEA | Precipitation seasonality | % | 1970-2000 | https://biogeo.ucdavis.edu/data/worldclim/v2.1/base/wc2.1_30s_bio.zip | 1 km |
| PWaQ | Precipitation of warmest quarter | mm | 1970-2000 | https://biogeo.ucdavis.edu/data/worldclim/v2.1/base/wc2.1_30s_bio.zip | 1 km |
| PCoQ | Precipitation of coldest quarter | mm | 1970-2000 | https://biogeo.ucdavis.edu/data/worldclim/v2.1/base/wc2.1_30s_bio.zip | 1 km |
| **EDAPHIC FACTORS (n = 19)** | | | | | |
| BD | Bulk density | kg m^-3^ | 1960-2015 | <https://files.isric.org/public/afsis250m/af_BLD_T__M_sd1_250m.tif> | 250 m |
| CEC | Cation exchange capacity | cmol kg^-1^ | 1960-2015 | <https://files.isric.org/public/afsis250m/af_CEC_T__M_sd1_250m.tif> | 250 m |
| NTOT | Total nitrogen | g kg^-1^ | 1960-2015 | https://files.isric.org/public/afsis250m/af_NTO_T__M_xd1_250m.tif | 250 m |
| SOC | Soil organic carbon | g kg^-1^ | 1960-2015 | <https://files.isric.org/public/afsis250m/af_ORCDRC_T__M_sd1_250m.tif> | 250 m |
| PH | pH in water |  | 1960-2015 | https://files.isric.org/public/afsis250m/af_PHIHOX_T__M_sd1_250m.tif | 250 m |
| SAND | Sand fraction | g 100g^-1^ | 1960-2015 | https://files.isric.org/public/afsis250m/af_SNDPPT_T__M_sd1_250m.tif | 250 m |
| BO | Extractable boron | mg kg^-1^ | 1960-2015 | <https://files.isric.org/public/af250m_nutrient/af250m_nutrient_b_m_agg30cm.tif> | 250 m |
| CA | Extractable calcium | mg kg^-1^ | 1960-2015 | <https://files.isric.org/public/af250m_nutrient/af250m_nutrient_ca_m_agg30cm.tif> | 250 m |
| COPPER | Extractable copper | mg kg^-1^ | 1960-2015 | <https://files.isric.org/public/af250m_nutrient/af250m_nutrient_cu_m_agg30cm.tif> | 250 m |
| FE | Extractable iron | mg kg^-1^ | 1960-2015 | <https://files.isric.org/public/af250m_nutrient/af250m_nutrient_fe_m_agg30cm.tif> | 250 m |
| MN | Extractable manganese | mg kg^-1^ | 1960-2015 | <https://files.isric.org/public/af250m_nutrient/af250m_nutrient_mn_m_agg30cm.tif> | 250 m |
| MG | Extractable magnesium | mg kg^-1^ | 1960-2015 | <https://files.isric.org/public/af250m_nutrient/af250m_nutrient_mg_m_agg30cm.tif> | 250 m |
| PHOS | Extractable phosphorus | Mg kg^-1^ | 1960-2015 | <https://files.isric.org/public/af250m_nutrient/af250m_nutrient_p_m_agg30cm.tif> | 250 m |
| POT | Extractable potassium | mg kg^-1^ | 1960-2015 | <https://files.isric.org/public/af250m_nutrient/af250m_nutrient_k_m_agg30cm.tif> | 250 m |
| SOD | Extractable sodium | mg kg^-1^ | 1960-2015 | <https://files.isric.org/public/af250m_nutrient/af250m_nutrient_na_m_agg30cm.tif> | 250 m |
| ZINC | Extractable zinc | mg kg^-1^ | 1960-2015 | <https://files.isric.org/public/af250m_nutrient/af250m_nutrient_zn_m_agg30cm.tif> | 250 m |
| AL | Extractable aluminium | mg kg^-1^ | 1960-2015 | <https://files.isric.org/public/af250m_nutrient/af250m_nutrient_al_m_agg30cm.tif> | 250 m |
| GEOAGE | Geological age based on surface geology |  | 1960-2000 | https://web.archive.org/web/20170519221949/http://worldgrids.org/doku.php/wiki:geaisg3 | 1 km |
| PLFU | Physiographic landform units |  | 2000-2002 | https://web.archive.org/web/20170519221949/http://worldgrids.org/doku.php/wiki:l3pobi3 | 1 km |
| **SOCIOECONOMIC FACTORS (n = 6)** | | | | | |
| GACCESS | Global accessibility | Hours to major cities | 2000 | <https://web.archive.org/web/20170519221949/http://worldgrids.org/doku.php/wiki:gacgem> | 1 km |
| GLC2K | Global land cover map |  | 2000 | <https://web.archive.org/web/20170519221949/http://worldgrids.org/doku.php/wiki:glcjrc3> | 1 km |
| MA500K | Market access of 500,000 people | Hours to market 500k people | 2006-2016 | <https://dataverse.harvard.edu/file.xhtml?persistentId=doi:10.7910/DVN/YKDWJD/UFOGZS&version=2.2> | 1 km |
| POPDEN | Population density | People km-2 | 2016 | <https://data.worldpop.org/GIS/Population_Density/Global_2000_2020_1km/2016/UGA/uga_pd_2016_1km.tif> | 1 km |
| UCD | Cattle density | Cattle km-2 | 2010 | <http://www.fao.org/ag/againfo/resources/en/glw/home.html> | 10 km |
| UPOV | Poverty index |  | 2011 | <https://data.worldpop.org/GIS/Development_and_health_indicators/Individual_countries/Poverty/UGA/79.7z> | 1 km |
| **TOPOGRAPHIC FACTORS (n = 6)** | | | | | |
| MPISR | SAGA Mean potential incoming solar radiation | kWh m^-2^ |  | [https://web.archive.org/web/20170519221949/http://worldgrids.org/doku.php/wiki:inmsre3](http://worldgrids.org/doku.php) | 1 km |
| SDPISR | SAGA Standard deviation of the potential incoming solar radiation | kWh m^-2^ |  | https://web.archive.org/web/20170519221949/http://worldgrids.org/doku.php/wiki:inssre3 | 1 km |
| STOI | SAGA Topographic openness index |  |  | https://web.archive.org/web/20170519221949/http://worldgrids.org/doku.php/wiki:opisre3 | 1 km |
| STWI | SAGA Topographic wetness index |  |  | https://web.archive.org/web/20170519221949/http://worldgrids.org/doku.php/wiki:twisre3 | 1 km |
| SLOPE | Global relief model derived slope | % | 2002-2010 | https://web.archive.org/web/20170519221949/http:/worldgrids.org/doku.php/wiki:slpsrt3 | 1 km |
| SRTM | Digital elevation model at 90 m resolution | m |  | <http://srtm.csi.cgiar.org/> | 90 m |
| **VEGETATION FACTORS (n = 19)** | | | | | |
| EVI | Enhanced vegetation index |  | 2000-2018 | ftp://africagrids.net/250m/MOD13Q1/Version6/EVI/ | 250 m |
| FAPARav | Fraction of absorbed photosynthetically active radiation­ – average | μmol m^−2^ s^−1^ | 1998-2014 | <ftp://africagrids.net/1000m/SPOT/FAPAR/> | 1 km |
| FAPARstd | Fraction of absorbed photosynthetically active radiation­ – standard deviation | μmol m^−2^ s^−1^ | 1998-2014 | <ftp://africagrids.net/1000m/SPOT/FAPAR/> | 1 km |
| FAPARvar | Fraction of absorbed photosynthetically active radiation­ – variance | μmol m^−2^ s^−1^ | 1998-2014 | <ftp://africagrids.net/1000m/SPOT/FAPAR/> | 1 km |
| GPPALT | Gross primary productivity – average | g C m^−2^ yr^−1^ | 2000-2010 | <ftp://africagrids.net/1000m/MOD17A3/GPP/> | 1 km |
| GPPVLT | Gross primary productivity – variance | g C m^−2^ yr^−1^ | 2000-2010 | <ftp://africagrids.net/1000m/MOD17A3/GPP/> | 1 km |
| LAIALT | Leaf area index – average |  | 2000-2017 | <ftp://africagrids.net/1000m/MOD15A2/Leaf_area_index/> | 1 km |
| LAISLT | Leaf area index – standard deviation |  | 2000-2017 | <ftp://africagrids.net/1000m/MOD15A2/Leaf_area_index/> | 1 km |
| LAIVLT | Leaf area index – variance |  | 2000-2017 | <ftp://africagrids.net/1000m/MOD15A2/Leaf_area_index/> | 1 km |
| LTEVAPO | Long-term MODIS evapotranspiration | mm yr^−1^ | 2000-2012 | https://web.archive.org/web/20170620001345/http://www.worldgrids.org/doku.php/wiki:etmnts3 | 1 km |
| MM8DLAI | Mean 8-day MODIS leaf area index | % | 2001-2012 | https://web.archive.org/web/20170620003514/http://www.worldgrids.org/doku.php/wiki:lammod3 | 1 km |
| MMMODISEVI | Mean monthly MODIS enhanced vegetation index |  | 2001-2012 | [https://web.archive.org/web/20170621190850/http://www.worldgrids.org/doku.php/wiki:evmmod3](https://web.archive.org/web/20170621190850/http:/www.worldgrids.org/doku.php/wiki:evmmod3) | 1 km |
| NDVI | Normalized difference vegetative index |  | 2000-2018 | <ftp://africagrids.net/250m/MOD13Q1/Version6/NDVI/> | 250 m |
| RB1RED | Red reflectance (Band 1) | nm | 2000-2018 | <ftp://africagrids.net/250m/MOD13Q1/Version6/Reflectance_red/> | 250 m |
| RB2NIR | Near infrared reflectance (Band 2) | nm | 2000-2018 | <ftp://africagrids.net/250m/MOD13Q1/Version6/Reflectance_NIR/> | 250 m |
| RB3BLUE | Blue reflectance (Band 3) | nm | 2000-2018 | <ftp://africagrids.net/250m/MOD13Q1/Version6/Reflectance_blue/> | 250 m |
| RB7MIR | Mid infrared reflectance (Band 7) | nm | 2000-2018 | <ftp://africagrids.net/250m/MOD13Q1/Version6/Reflectance_MIR/> | 250 m |
| SD8LAI | 8-day MODIS Leaf area index – standard deviation | % | 2001-2012 | https://web.archive.org/web/20170620001518/http://www.worldgrids.org/doku.php/wiki:lasmod3 | 1 km |
| SDMEVI | Monthly MODIS enhanced vegetation index – standard deviation |  | 2001-2012 | https://web.archive.org/web/20170620003558/http://www.worldgrids.org/doku.php/wiki:evsmod3 | 1 km |

*MODIS – Moderate Resolution Imaging Spectroradiometer; SAGA – System for Automated Geoscientific Analyses
